# Maternal and Paternal Dietary Quality and Dietary Inflammation Associations with Offspring DNA Methylation and Epigenetic Biomarkers of Aging in the Lifeways Cross-Generation Study

**DOI:** 10.1016/j.tjnut.2023.01.028

**Published:** 2023-01-28

**Authors:** Marion Lecorguillé, Pilar Navarro, Ling-Wei Chen, Celine Murrin, Karien Viljoen, John Mehegan, Nitin Shivappa, James R. Hébert, Cecily C. Kelleher, Matthew Suderman, Catherine M. Phillips

**Affiliations:** 1 School of Public Health, Physiotherapy and Sports Science, University College Dublin, Dublin, Ireland; 2 Institute of Epidemiology and Preventive Medicine, College of Public Health, National Taiwan University, Taipei, Taiwan; 3 Cancer Prevention and Control Program and Department of Epidemiology and Biostatistics, Arnold School of Public Health, University of South Carolina, Columbia, SC, USA; 4 Connecting Health Innovations, LLC, Columbia, SC, USA; 5 MRC Integrative Epidemiology Unit, Population Health Sciences, Bristol Medical School, University of Bristol, Bristol, United Kingdom

**Keywords:** parental dietary quality, Healthy-Eating Index (HEI), Dietary Inflammatory Index (DII), DNA methylation, epigenetic biomarkers of aging

## Abstract

**Background:**

Early-life nutritional exposures may contribute to offspring epigenetic modifications. However, few studies have evaluated parental dietary quality effects on offspring DNA methylation (DNAm).

**Objectives:**

We aim to fill this gap by elucidating the influence of maternal and paternal whole-diet quality and inflammatory potential on offspring DNAm in the Lifeways Cross-generation cohort.

**Methods:**

Families (*n* = 1124) were recruited around 16 weeks of gestation in the Republic of Ireland between 2001 and 2003. Maternal dietary intake during the first trimester and paternal diet during the 12 previous months were assessed with an FFQ. Parental dietary inflammatory potential and quality were determined using the energy-adjusted Dietary Inflammatory Index (E-DII), the Healthy Eating Index-2015 (HEI-2015), and the maternal DASH score. DNAm in the saliva of 246 children at age nine was measured using the Illumina Infinium HumanMethylationEPIC array. DNAm-derived biomarkers of aging, the Pediatric-Buccal-Epigenetic clock and DNAm estimator of telomere length, were calculated. Parental diet associations with the DNAm concentrations of 850K Cytosine-phosphate-guanine sites (CpG sites) and with DNAm-derived biomarkers of aging were examined using an epigenome-wide association study and linear regressions, respectively.

**Results:**

Maternal HEI-2015 scores were inversely associated with DNAm at CpG site (cg21840035) located near the *PLEKHM1* gene, whose functions involve regulation of bone development (β = −0.0036, per 1 point increase in the score; *P* = 5.6 × 10^−8^). Higher paternal HEI-2015 score was related to lower methylation at CpG site (cg22431767), located near cell signaling gene *LUZP1* (β = −0.0022, per 1 point increase in the score, *P* = 4.1 × 10^−8^). There were no associations with parental E-DII and DASH scores, and no evidence of major effects on biomarkers of aging.

**Conclusions:**

Parental dietary quality in the prenatal period, evaluated by the HEI-2015, may influence offspring DNAm during childhood. Further research to improve our understanding of parental nutritional programming is warranted.

## Introduction

Adequate nutritional status is essential for a healthy pregnancy and optimal fetal growth. Barker et al. [1] suggested that the prenatal environment might influence the child’s development and susceptibility to chronic diseases in adulthood, a concept now known as the *developmental origins of health and disease* [1]. However, the biological mechanisms underlying this link have not been fully elucidated. Epigenetic mechanisms (that is, changes in gene function not mediated by DNA nucleotide sequence alteration), particularly DNA methylation (DNAm), may play a role by responding to and retaining a long-term memory of the *in utero* environment [2,3]. Increasing evidence indicates that DNAm is labile in response to nutritional and environmental influences. However, so far, only few studies have investigated the extent to which parental diet influences the offspring epigenome.

In recent years, there is growing evidence supporting the overall diet quality as a more effective approach for assessing health impacts and developing public health prevention than the examination of individual foods or nutrients [4]. Dietary quality may be ascertained by scoring adherence to dietary guidelines [the Healthy Eating Index (HEI)] ([5,6]; by evaluating the adherence to a specific dietary pattern such as the Mediterranean diet [7] and the DASH [8]; and scoring dietary patterns known to be associated with health such as the Dietary Inflammatory Index (DII) [9]. The DII is of particular interest for pregnancy as chronic inflammation is associated with a variety of adverse outcomes [10]. A recent meta-analysis of 16 295 to 24 861 mother–child pairs showed that a maternal diet of low quality or high inflammatory potential was associated with altered fetal growth and offspring obesity risk [11,12].

Analogously to diet, associations with DNAm can be investigated at individual genomic loci or more holistically with respect to indices of DNAm derived from multiple loci across the genome. In recent years, DNAm-derived indices that serve as a proxy for aging and telomere length (TL) have been described, and evidence suggests links to prenatal exposures, including nutrition, with early-life markers of chronic diseases during adulthood [13]. For example, individuals with accelerated aging estimates appear to have increased risk of all-cause mortality [14]. TL plays a fundamental role in maintaining genome and cell integrity, and most of the interindividual variation in adult TL is established in early life [15]. However, limited data regarding maternal dietary influence on offspring epigenetic biomarkers of aging exist. Furthermore, numerous animal models have provided evidence that the paternal environment plays a role in a nongenetic inheritance of preconceptional exposures through the male germ line [16]. There has been increasing interest in the epigenetic mechanisms through which paternal influences may affect the child development [17]. The Paternal Origins of Health and Disease paradigm was introduced to highlight the need to conduct research on the father’s role in the transmission of his environment to his offspring [16]. However, the impact of paternal diet on offspring DNAm is still relatively unknown [18,19].

We hypothesized that maternal and paternal dietary quality and dietary inflammatory status in the prenatal period may influence long-term DNAm of children. Using a genome-wide approach, we explored the association of parental dietary quality (HEI-2015 and maternal DASH scores) and dietary inflammation status (energy-adjusted DII [E-DII]), same for (HEI-2015) with offspring DNAm at 9 y of age at 850K CpG sites across the genome and with DNAm-derived biomarkers of aging (Pediatric-Buccal-Epigenetic clock (PedBE) and DNAm estimator of telomere length (DNAmTL)).

## Methods

### Study design

**FIGURE 1:**
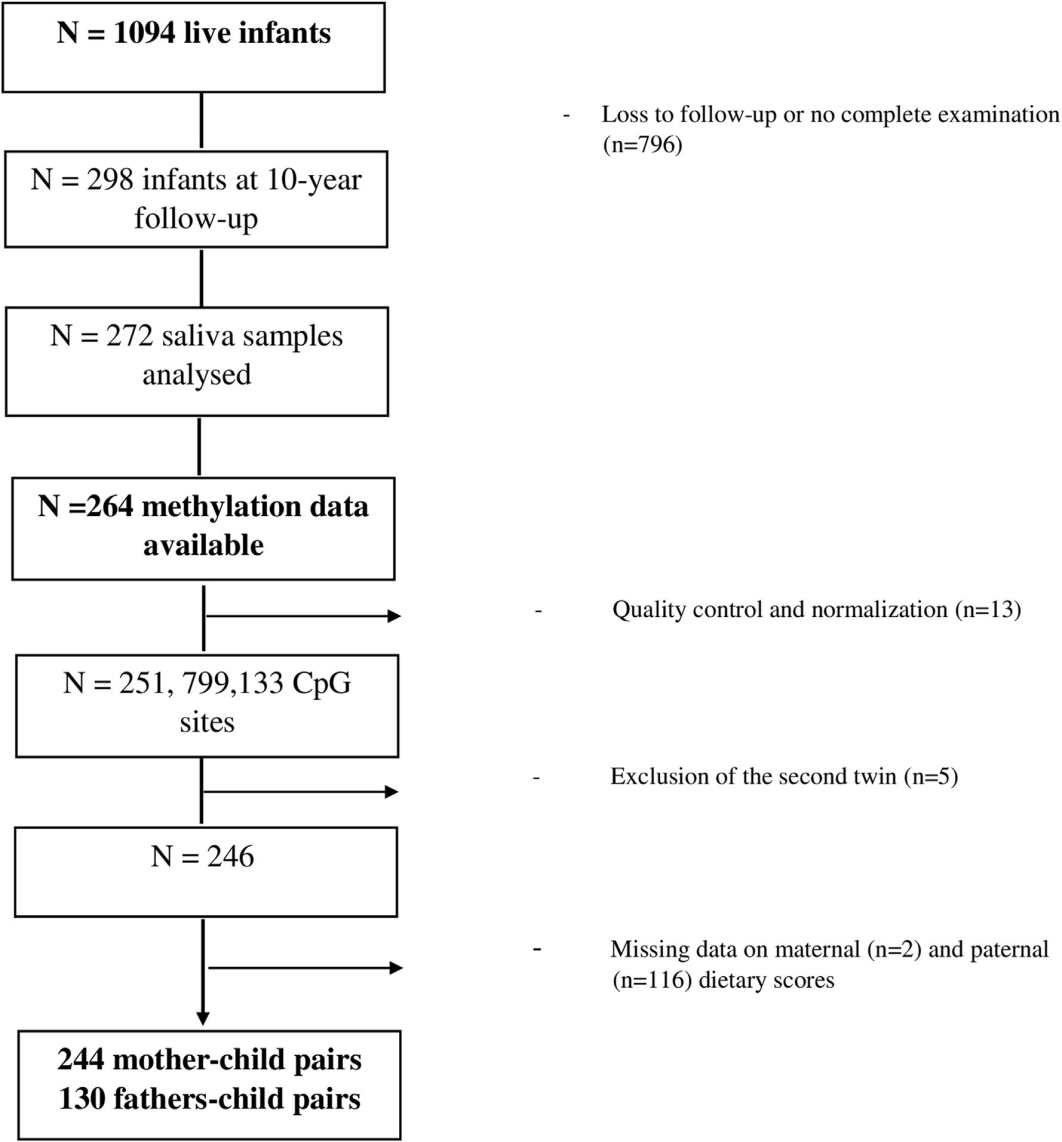
Flow-chart of population selection, Lifeways Cross-Generation Cohort Study, Dublin and Galway, Ireland, 2001–2012.

The Lifeways Cross-Generation Cohort Study is a prospective family study whose objectives are to document health status, diet, and lifestyle in the family members and to establish patterns and links across generations [20]. Mothers (*n* = 1124) were initially recruited by a midwife during their first antenatal visit at two maternity hospitals in the Republic of Ireland between 2001 and 2003. A cohort of 1094 live infants were born to these mothers including 12 pairs of twins (*n* = 1082 mothers). The participating mothers’ partners (that is, the biological fathers) were also directly contacted by the Lifeways research team at recruitment (participant mothers having given their contact details at their booking visit) and invited to participate (*n* = 333 agreed). Lifeways is a longitudinal cohort study with several waves of data collection since its establishment two decades earlier. The database of families is regularly updated and contact maintained with periodic newsletters and other communications. There have been relatively few formal withdrawals from the study. Longitudinal follow-up was conducted with linkage data to hospital and general practice records and examination of children when aged on average 5 and 9 y. During 2012, examinations of height and weight of children (*n* = 298) were conducted through general practitioners and saliva samples were collected. All participants gave their informed consent for inclusion before participation in the study. We do not have data for other families because of loss to follow-up and incomplete examination, but we believe that the practical logistics of attending appointments may have influenced the response rates, rather than any systematic bias in follow-up. Despite loss to follow-up, we can highlight a substantial percentage of children at follow-up with epigenetic data (24.9%), high percentage of their fathers included (53%), and representativeness of the children in terms of their mothers characteristics [20]. Ethical approval was granted by the ethics committees of the Coombe Women’s and Infants University Hospital, Dublin, University College Dublin, Irish College of General Practitioners, and University College Hospital, Galway, Ireland. Written informed consent was collected from all women upon recruitment and to all subsequent steps of the study. DNA was extracted and quantified from 264 offspring saliva samples collected at 9-y follow-up visit and DNAm was measured. These included five sets of twins. As twins share genetic factors, we retained one out of each twin pair. Following the DNAm quality control and normalization steps (detailed below), exclusion of missing data on dietary scores, 244 mother–child pairs and 130 father–child pairs were included in the analyses. The selection of the population is presented in Figure 1. Mothers and fathers completed a questionnaire with sections relating to general health, smoking, and sociodemographic characteristics. Habitual dietary intakes of the women during the first trimester of pregnancy were assessed at inclusion (12–16 weeks of gestation) using a validated 149-item, semiquantitative FFQ [21]. Fathers also completed the same FFQ at baseline, providing information on their habitual diet for the last year.

### Dietary assessment

Briefly, a modified version of the self-completed *European Prospective Investigation* into Cancer and Nutrition FFQ was used [22] and adapted to reflect the Irish diet [23]. The version used was originally validated using food diaries and a protein biomarker in a volunteer sample and then incorporated into the Irish survey of lifestyle attitude and nutrition 1998, 2002, 2006. The FFQ was also validated using a 7-d weighed food record, completed by a subsample of the Lifeways mothers at 5-y follow-up visit, with reasonable agreement (*r* > 0.40) for fat, carbohydrate, and their components, and with low agreement for protein (*r* = 0.25) [24]. Participants were asked about their average consumption frequency (9 levels, *from never or less than once per month to ≥6 times per day*) of each food item. The daily quantities of food intakes were then derived by multiplying the frequencies per day with standard portion sizes [25]. Daily energy and nutrient intakes were computed for each participant using a software program (FFQ Software Ver 1.0; developed by the National Nutrition Surveillance Centre, University College Dublin, Ireland) based on the McCance and Widdowson Food Tables [25].

#### Dietary quality scores

Parental dietary quality was assessed by the HEI-2015 scores, which is the most recent iteration based on conformance with the 2015–2020 Dietary Guidelines for Americans [6,26]. The HEI-2015 contains 13 components, which are scored on a density basis out of 1000 kcal, with the exception of fatty acids, which is a ratio of unsaturated to SFAs [6,27]. Absolute scores below 51 indicate a *poor* diet, scores between 51 and 80 correspond to “needing dietary improvements,” whereas scores above 80 indicate a *good* diet [28]. The food components, calculation, and interpretation of the HEI scores are presented in Supplemental Table 1.

Dietary quality was also assessed for the mothers by their degree of adherence to the DASH diet. The DASH diet is widely promoted in particular for the prevention and treatment of hypertension [29] and is included as an example of a healthy eating pattern in the 2005 Dietary Guidelines for Americans. DASH score generation has been described elsewhere [8,11]. In brief, in the Lifeways study 85 FFQ food items (excluding alcohol) were used for creating the DASH scoring criteria [11]. The DASH score was generated based on the index proposed by Fung et al. [29], which aims to classify women into quintiles according to their intake ranking and represents a relative adherence measure adapted to our population. The DASH score comprised eight food components. Supplemental Table 1 presents the description of the DASH. A high DASH score reflects an increase in the dietary quality. The DASH score was not available for fathers.

#### DII

Parental dietary inflammatory potential was determined using the energy-adjusted DII, a validated literature-derived score from the original DII, calculation of which has been previously described in detail [30]. Defined by Shivappa et al. [30], the DII combines a range of macronutrients and micronutrients which either increased or decreased the concentrations of circulating biomarkers of inflammation (C-reactive protein, IL1-β, IL-4, IL-6, IL-10, and TNFα) [30] (Supplemental Table 2). The original DII is based on 45 food parameters. However, on average, 28 food parameters are used for DII generation, as is the case here, and comparative analysis has shown no change in the predictive ability of the DII when going from 45 to <30 food parameters [31,32]. The DII was designed to be universally applicable across all human studies with adequate dietary assessment [33]. However, the observation that the relationship between DII and energy intake is complex and differs across data led to the development of the E-DII [33]. Calculation of the E-DII in the Lifeways study has been described in detail elsewhere [11,31]. A high E-DII score indicates a high proinflammatory diet.

### Classification of variables

At recruitment, mothers provided information on age, self-reported height, and prepregnancy weight (from which prepregnancy BMI was calculated), education level (below tertiary compared with tertiary or above (no schooling/primary/secondary compared with third-level education); when self-reported data were missing, information was reported by the partner). Data were also collected regarding household total net income per week (in three categories: <£ 200, £ 200–£ 600, >£ 600), marital status (married/cohabiting compared with divorced/widowed/single), and socioeconomic status (proxied by eligibility to the General Medical Services Scheme, a robust indicator of social disadvantage in Ireland) [34]. Smoking status during pregnancy (yes/no) was also assessed using the same questionnaire at baseline and from the mother’s hospital record (a composite variable with information of current smokers, smoking status at booking or at delivery). Paternal current smoking status at recruitment (consumption of cigarettes, cigars, or pipes) was recorded. Additional information related to pregnancy including parity, child sex, gestational age at birth, and birthweight (in grams) was selected from the mother’s hospital record.

### DNA methylation assessment

Saliva samples of children were collected into Genotek Oragene tubes (DNA Genotek Inc.) during a medical examination, when the children were 9.8 y on average, performed by general practitioners. DNA was extracted following the Genotek Oragene saliva DNA extraction protocol using a PerkinElmer Prepito-D automated machine (PerkinElmer Inc.). Extracted DNA was quantified by a manual flurometric assay using picogreen reagent. Samples with low DNA concentration or quality based on the A260:280 ratio (<1.8) were excluded from further analysis. Bisulphite conversion of DNA samples (500 ng) was conducted using the Zymo EZ-96 Methylation kit (Zymo Research) according to the manufacturer’s instructions. The Illumina Infinium HumanMethylation EPIC BeadChip assay (Illumina 850 K array) was used to evaluate the methylation status of 865 859 individual CpG sites in the saliva samples (*n* = 264) (Illumina Inc.). Arrays were scanned using an Illumina iScan and control software. Hybridization/fluorescence signals were written to .IDAT files, and initial quality review was assessed using GenomeStudio at the Bristol Bioresource Laboratories.

Quality control and normalization of the bead chip data was performed using the *meffil* R package [35]. For quality control, we checked for variation caused by technical artifacts, extreme dye-bias, ratio of unmethylated-to-methylated signal outliers, control probe outliers, and probes and samples with low signal-to-background ratios. We excluded samples showing evidence of extreme dye-bias and outliers on bisulfite conversion, and sample mismatch (*n* = 13). We excluded probes targeting the sex chromosomes, probes with a bead count <3 in >10% of samples, and probes having a detection *P* value >0.01 in >10% of samples, which led to 251 samples and 799,133 CpG sites measured for analyses. Samples were normalized by using functional normalization as implemented in the *meffil* R package. This includes a method to identify the number of control probe principal components that minimizes residual variance within a cross-validation framework. This parameter was set to 15 [35]. Probe intensity quantiles were then normalized by removing variance of the principal components as fixed effects. Normalized methylation levels for each sample are then derived from the normalized quantiles. Methylation levels or β-values at CpG sites were estimated as ratios of the methylated probe intensity to the overall probe intensity (sum of methylated and unmethylated probe signals) ranging from 0 to 1. Saliva is known to have a heterogeneous cellular composition, mostly composed of buccal epithelial cells and leukocytes. In the absence of saliva cell type reference methylomes, *we used* a combined set of references obtained from blood leukocytes and buccal cells to estimate the cellular composition in saliva DNAm using the Houseman deconvolution method [36].

### Statistical analyses

Characteristics of the population with available data on dietary scores and methylation are described with mean ± SD and numbers (%). We also compared characteristics between families followed-up until 10 y with available epigenetic data and those not included in the analyses by chi-squared test for categorical variables and ANOVA for continuous variables.

#### Epigenome-wide association study

Using the *meffil* package, we performed epigenome-wide association studies (EWAS) to identify the associations between each of the dietary scores (HEI-2015, E-DII, DASH) and DNAm at individual CpG sites. The associations were adjusted for potential confounders identified from the available literature, and technical factors related to methylation measurement. Three models were applied. Model (1) was considered as the *main model* and included adjustment for child sex, parental smoking status, batch effect (represented by the sample plate variable), and estimated cell-type proportions. Counts of seven cell-types were estimated in the saliva tissue (Buccal, B cell, granulocytes, natural killer cells, monocytes, CD4+ T cell, CD8+ T cell) [35]. Low counts were estimated for all cell types except *buccal*, *B cell*, *granulocytes*, and *natural killer cells* in analyses, so only these four were included in the model. Model (2) included further adjustments for maternal age (continuous), education level, parity, and birthweight (continuous) (these factors have been described as associated with methylation [37,38]). Model (3) included further adjustment for maternal BMI and gestational age (continuous variables). All analyses were performed on samples with complete data. Models are described in Supplemental Table 2. Similar models were used for evaluating the effect of father’s diet. As the DASH score was not intrinsically adjusted for energy intake, models for DASH scores were additionally adjusted for energy intake. Sensitivity analyses were also conducted excluding mothers and fathers with implausible energy intakes to avoid extreme misreporting by considering a general cut-off of <500 or >5000 kcal/d, and gender specific stricter cut-offs of <500 or >3500 kcal/d for mothers [39,40] and <800 or >4200 kcal/d for fathers [41]. We used Bonferroni correction to derive a conservative *P* value threshold of 6.3 × 10^−8^ (*P* < 0.05 for 799 133 independent tests) to identify CpG sites strongly associated with the dietary scores [42]. We also tested whether the strengths of the associations differed between maternal and paternal models comparing the top CpG associations. A similar approach was used to compare the HEI and E-DII effects on child DNAm. We tested the statistical significance considering the threshold of 0.0005 (0.05/number of tests for multiple correction).

Differentially methylated regions (DMRs) were identified using the *dmrff* package in R, which identifies DMRs by meta-analyzing EWAS summary statistics (from the *main model*) across all CpG sites in a genomic region while adjusting for the correlations among CpG sites [43]. A region was considered a DMR if the Bonferroni-adjusted *P* value for the region was <0.05. All statistical analyses were performed with R v3.6.0.

#### Epigenetic biomarkers of aging

##### Pediatric-Buccal-Epigenetic (PedBE) clock

DNAm-derived age for each child’s saliva sample was estimated by applying the recently described Pediatric-Buccal-Epigenetic (PedBE) clock method [44]. The PedBE method was developed using DNAm from the buccal epithelial cells collected from individuals aged between 0 and 20 y and found to be a highly accurate measure of epigenetic age in the pediatric population [44]. Because there is a large predominance of buccal epithelial cells in saliva samples, especially in young children, it is appropriate for use with saliva DNAm [45]. Accelerated aging was estimated as the residual from the linear regression of the PedBE estimate on chronological age, additionally adjusted for the four major estimated cell-types and batch (plate). Positive values indicate accelerated aging and negative values indicate decelerated aging. Children with missing values on exact age at methylation measurement were therefore excluded (*n* = 5) from analyses. Using linear regression models, associations of parental diet with the accelerated aging (in years) were assessed. Similar models as described previously for EWAS were fitted.

##### DNA methylation-based estimator of telomere length

Offspring telomere length was estimated using a DNAmTL [46] implemented by the online Horvath DNAm age calculator (https://dnamage.genetics.ucla.edu/). The DNA methylation and sample annotation files were used as input with the *normalize data* option selected. Accelerated telomere aging was estimated as the residuals obtained by regressing DNAmTL on the current age of children and adjusting for batch (sample plate) and cell-type proportions. Associations with accelerated telomere aging were tested using the EWAS models previously described.

## Results

### Population characteristics

The characteristics of the families selected for analysis are summarized in Table 1. Mean maternal and paternal age was 32 (±4.9) and 34 (±5.1) y, respectively. Most mothers and fathers (61% and 58.5%, respectively) had a tertiary education level, and 42.5% of women had a weekly household income >£600. Approximately 18% of mothers smoked during pregnancy, and 37.5 % of fathers were current smokers. On average, the maternal HEI-2015 scores show that women’s diet *needs improvement* [mean ± SD was 53.4 ± 7.8 (range: 34–77)]; and fathers had a *poor diet* [48.3 ± 8.6 (range: 30–71)]. Fathers presented a more proinflammatory diet than mothers [E-DII mean ± SD was 0.11 ± 1.6 for mothers (range: −4 to 5) compared with 1.4 ± 1.7 (range: −3 to 5)]. The mean ± SD of maternal DASH scores was 24.7 ± 4.4 (range, 14–35). The comparison of characteristics at 10-y follow-up between families who had epigenetic data and those who did not is presented in Supplemental Table 3. Families included in the current analysis had higher education and income levels as well as were less likely to be smokers or to have a medical card than families not followed-up.

**TABLE 1:** Characteristics of the study population, Lifeways Cross-Generation Cohort Study, Dublin and Galway, Ireland, 2001–2012

Family characteristics	Missing data *n* (%)	Population
Maternal characteristics (*n* = 244)		
Maternal age (y)	0 (0)	31.9 ± 4.9
Maternal education	0 (0)	
Below tertiary		96 (39.3)
Tertiary or above		148 (60.7)
Medical card (yes)	2 (0.8)	24 (9.9)
Marital status	0 (0)	
Married/cohabiting		216 (88.5)
Separated/divorced/single		28 (11.5)
Household weekly income	18 (7.4)	
< £200		11 (4.9)
£200–£600		119 (52.7)
>£600		96 (42.5)
Maternal smoking (yes)	0 (0)	44 (18)
Primiparous	3 (1.2)	100 (41.5)
Prepregnancy BMI (kg/m^2^)	31 (12.7)	23.4 ± 3.3
Maternal E-DII	0 (0)	0.11 ± 1.6
Maternal HEI-2015	0 (0)	53.4 ± 7.8
Maternal DASH	0 (0)	24.7 ± 4.4
Offspring characteristics (*n* = 244)		
Child sex	0 (0)	
Male		114 (46.7)
Female		130 (53.3)
Birthweight (grams)	0 (0)	3503.6 ± 599.6
Gestational age at birth (wk)^1^	17 (7.0)	39.9 ± 2.0
Paternal characteristics (*n* = 130)		
Paternal age (y)	0 (0)	34.37 ± 5.1
Paternal education	0 (0)	
Below tertiary		54 (41.5)
Tertiary or above		76 (58.5)
Paternal smoking (yes)	2 (0.8)	48 (37.5)
Paternal BMI (kg/m^2^)	14 (10.8)	26.7 ± 3.6
Paternal E-DII	0 (0)	1.37 ± 1.7
Paternal HEI-2015	0 (0)	48.3 ± 8.6

Values are means ± SD for continuous variables and *n* (%) for categorical variables. BMI, body mass index; E-DII, energy-adjusted dietary inflammatory index; HEI, healthy eating index; DASH, dietary approach to stop hypertension. ^1^Weeks, weeks of gestation.

### Epigenome-wide association studies of diet indices

**FIGURE 2:**
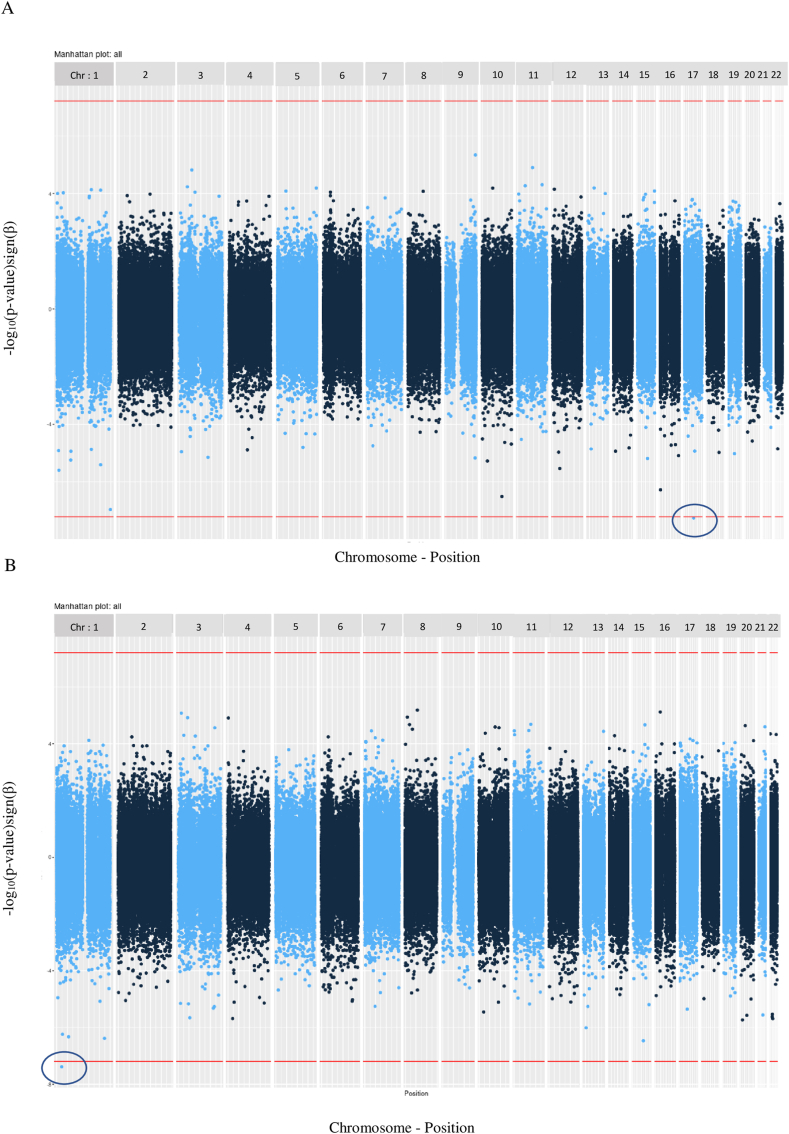
Manhattan plot of epigenome-wide association results in offspring associated with the maternal (A) and paternal (B) Healthy Eating Index-2015. The red dashed line represents Bonferroni correction significance threshold (*P* = 6.3 × 10^−8^). The direction of the associations is reported with negative effects below zero and positive effects above zero. Model adjusted for batch effect, child sex, maternal or paternal smoking, and cellular composition.

Manhattan plots showing associations with maternal and paternal HEI-2015 scores are presented in Figure 2A, B. It provides a graphic representation of the distribution of CpG site associations.

Maternal HEI-2015 scores were inversely associated with DNAm at one CpG site (cg21840035) located near the *PLEKHM1* gene (Pleckstrin Homology Domain-Containing Family M Member 1) (β = −0.0036, 95% CI: −0.0049; −0.0024 per 1 point increase in the score; *P* = 5.6 × 10^−8^; adjusted *P* = 0.04). Table 2 shows the 10 most strongly associated CpG sites with each maternal dietary score from the main model. After adjustment for additional potential confounding variables, the association with maternal HEI-2015 score persisted with similar effects (*P* value in model 2 = 7.4 × 10^−8^ and *P* value in model 3 = 3.6 × 10^−8^) (see Supplemental Table 4). No associations between maternal E-DII or DASH scores and child DNAm survived the adjustment for multiple tests. In sensitivity analyses excluding women with implausible energy intakes, the association between maternal HEI-2015 score and child DNAm was slightly attenuated but effects remained similar (β = −0.0036, *P* value = 1.3 × 10^−7^ and β = −0.0032, *P* value = 8.1 × 10^−6^ for <500 or >5000 kcal/d and <500 or >3500 kcal/d, respectively) (see Supplemental Table 5).

**TABLE 2:** Associations between maternal dietary scores and the top 10 individual CpG sites (main model) in the Lifeways Cross-Generation Cohort Study (*n* = 244)

	Probe ID	β (95% CI)^1^	*P* value	Chromosome	Gene symbol	Gene region	Relation to CpG island	Common SNP^2^
Maternal E-DII								
	cg20748132	0.015 (0.0095, 0.0206)	2.28E-07	chr12				FALSE
	cg00109781	−0.007 (−0.0098, −0.0042)	1.47E-06	chr9				TRUE
	cg13993877	−0.0153 (−0.0214, −0.0092)	1.47E-06	chr1	PRDM16	Body	N_Shore	TRUE
	cg26871350	0.0114 (0.0067, 0.016)	2.60E-06	chr5	F2RL2; IQGAP2	TSS1500; Body	OpenSea	TRUE
	cg26381263	−0.0133 (−0.0189, −0.0078)	4.03E-06	chr10	PPP2R2D	TSS200	N_Shelf	FALSE
	cg22070649	0.0095 (0.0055, 0.0134)	5.05E-06	chr2				FALSE
	cg06708956	0.0109 (0.0062, 0.0155)	6.47E-06	chr4				FALSE
	cg01488575	0.0083 (0.0048, 0.0119)	7.73E-06	chr8				TRUE
	cg14336308	−0.013 (−0.0185, −0.0074)	7.96E-06	chr22	CRELD2	Body	N_Shore	TRUE
	cg24284539	0.0249 (0.0142, 0.0356)	8.05E-06	chr10	CCDC3	Body	OpenSea	TRUE
Maternal HEI-2015								
	cg21840035	−0.0036 (−0.0049, −0.0024)	5.56E-08	chr17	PLEKHM1	5′UTR;Body	N_Shore	TRUE
	cg15478184	−0.003 (−0.0041, −0.0019)	1.10E-07	chr1	OR2L3;OR2L13	TSS1500;5′UTR	OpenSea	TRUE
	cg04776779	−0.0022 (−0.003, −0.0014)	3.10E-07	chr10				FALSE
	cg01455766	−0.0039 (−0.0054, −0.0024)	5.27E-07	chr16				TRUE
	cg06199676	−0.0025 (−0.0035, −0.0015)	2.55E-06	chr1				FALSE
	cg22082469	−0.0021 (−0.0029, −0.0012)	2.91E-06	chr12				FALSE
	cg05437285	−0.0029 (−0.0041, −0.0017)	3.92E-06	chr1	SYT2	5′UTR	OpenSea	FALSE
	cg00109781	0.0014 (0.0008, 0.002)	4.57E-06	chr9				TRUE
	cg11468003	−0.0027 (−0.0038, −0.0016)	5.30E-06	chr10				FALSE
	cg04839673	−0.0015 (−0.0022, −0.0009)	5.76E-06	chr1	PGM1	Body	OpenSea	TRUE
Maternal DASH								
	cg15119693	−0.0035 (−0.0048; −0.0021)	9.00E-07	chr8	EPHX2	Body	OpenSea	FALSE
	cg10210739	0.0023 (0.0014; 0.0033)	2.55E-06	chr1	S1PR1	5′UTR	S_Shore	TRUE
	cg08661219	0.0051 (0.0029; 0.0072)	5.78E-06	chr5				FALSE
	cg20095560	−0.004 (−0.0058; −0.0023)	9.47E-06	chr19	VS.TM2B	3′UTR	OpenSea	TRUE
	cg20552468	0.0054 (0.003; 0.0077)	1.41E-05	chr17	SLC38A10	Body	S_Shore	TRUE
	cg17859359	−0.0024 (−0.0034; −0.0013)	1.42E-05	chr1				FALSE
	cg25364619	−0.0066 (−0.0095; −0.0037)	1.45E-05	chr17	NXN	Body	OpenSea	FALSE
	cg22806934	−0.003 (−0.0043; −0.0016)	1.81E-05	chr11	UBASH3B	Body	OpenSea	FALSE
	cg17746360	−0.0075 (−0.0108; −0.0041)	1.91E-05	chr19	ARID3A	Body	OpenSea	TRUE
	cg18045100	−0.0029 (−0.0041; −0.0016)	2.01E-05	chr5				FALSE

Model adjusted for batch effect, child sex, maternal smoking, and cellular composition. Additional adjustment for energy intake considered for the DASH score. E-DII, energy-adjusted dietary inflammatory index; HEI, Healthy Eating Index; SNP, single-nucleotide polymorphisms. ^1^Values are increase of methylation expressed per unit increase in the E-DII and per 1 point increase in the HEI-score as well as DASH score. ^2^Single-nucleotide polymorphisms equal to TRUE for any probes that contain a common single-nucleotide polymorphism with minor allele frequency > 0.01.

An increase in paternal HEI-2015 score was associated with low methylation at one CpG site (cg22431767) located near cell signaling gene *LUZP1* (Leucine Zipper Protein 1) (β = −0.0022, 95% CI: −0.003; −0.0015, *P* = 4.1 × 10^−8^, adjusted *P* = 0.03) (Figure 2B).

However, this association was slightly attenuated after additional adjustment (*P* value for models 2 and 3 were <1.06 × 10^−6^) (Supplemental Table 4). No associations between paternal E-DII scores and child DNAm survived the adjustment for multiple tests (Table 3). In sensitivity analyses excluding fathers with implausible energy intakes, associations between paternal HEI-2015 score and child DNAm were similar (β = −0.0022, *P* value = 4.4 × 10^−8^; β = −0.0022 and *P* = 1.8 × 10^−7^ for <500 or >5000 kcal/d and <800 or >4200 kcal/d, respectively) (see Supplemental Table 5). Results were similar using the false-discovery rate (FDR) correction. We also found that EWAS associations with paternal and maternal HEI-2015 persist when including all seven estimated cell-types in EWAS model 1 (data not shown). We also found that results for the paternal HEI-2015 association with child DNA methylation are similar when adjusting for the maternal dietary score, even if the p value was slightly attenuated following additional adjustment. This result highlights the independent effect of the paternal diet (data not shown).

**TABLE 3:** Associations between paternal dietary scores and the top 10 individual CpG sites (main model) in the Lifeways Cross-Generation Cohort Study (*n* = 127)

	Probe ID	β (95% CI)^1^	*P* value	Chromosome	Gene symbol	Gene region	Relation to CpG island	Common single-nucleotide polymorphisms^2^
Paternal E-DII								
	cg16918683	0.0178 (0.0112, 0.0243)	4.59E-07	chr15	IGF1R	Body	OpenSea	TRUE
	cg22431767	0.0103 (0.0065, 0.0142)	6.33E-07	chr1	LUZP1	5′UTR	OpenSea	TRUE
	cg26790423	0.0189 (0.0117, 0.026)	8.98E-07	chr2	FER1L5	Body	OpenSea	FALSE
	cg20916830	0.0268 (0.016, 0.0376)	3.40E-06	chr1				FALSE
	cg08287737	0.0174 (0.0103, 0.0244)	3.89E-06	chr2				TRUE
	cg07879720	0.0139 (0.0082, 0.0195)	4.47E-06	chr5	WDR70	Body	OpenSea	FALSE
	cg16898495	0.014 (0.0082, 0.0199)	7.83E-06	chr13				FALSE
	cg24285545	0.0086 (0.005, 0.0122)	9.79E-06	chr2				TRUE
	cg13400365	0.0157 (0.009, 0.0224)	1.22E-05	chr15	KLHL25	TSS1500	S_Shore	FALSE
	cg13374264	0.0138 (0.0078, 0.0197)	1.32E-05	chr1	CAMTA1	Body	OpenSea	FALSE
Paternal HEI-2015								
	cg22431767	−0.0022 (−0.003, −0.0015)	4.12E-08	chr1	LUZP1	5′UTR	OpenSea	TRUE
	cg15311954	−0.0038 (−0.0052, −0.0024)	3.43E-07	chr15	MIR548H4	Body	S_Shelf	TRUE
	cg18506400	−0.0029 (−0.0039, −0.0018)	4.16E-07	chr1	SUSD4	Body	OpenSea	FALSE
	cg14977608	−0.0034 (−0.0047, −0.0022)	4.73E-07	chr1	PCSK9	Body	S_Shore	TRUE
	cg20135776	−0.0019 (−0.0026, −0.0012)	5.81E-07	chr1	ARID1A	Body	OpenSea	FALSE
	cg20595323	−0.0028 (−0.0038, −0.0017)	9.79E-07	chr13	EEF1DP3	Body	OpenSea	FALSE
	cg08955721	−0.0019 (−0.0026, −0.0011)	1.85E-06	chr20	SNAP25-AS1	Body	N_Shore	FALSE
	cg14833293	−0.0029 (−0.004, −0.0017)	2.05E-06	chr22				TRUE
	cg03271761	−0.0036 (−0.0051, −0.0022)	2.06E-06	chr4	KCNIP4	5′UTR;1stExon;5′UTR	OpenSea	FALSE
	cg25618378	−0.0041 (−0.0057, −0.0025)	2.22E-06	chr3	CACNA1D	Body	OpenSea	FALSE

Model adjusted for batch effect, child sex, paternal smoking, and cellular composition. E-DII, energy-adjusted dietary inflammatory index; HEI, Healthy Eating Index. ^1^β-Values are expressed per unit increase in the E-DII, per 1 point increase in the HEI-score. ^2^Single-nucleotide polymorphism equal to TRUE for any probes that contain a common single-nucleotide polymorphisms with minor allele frequency >0.01.

#### The maternal compared with paternal effect of diet

**FIGURE 3:**
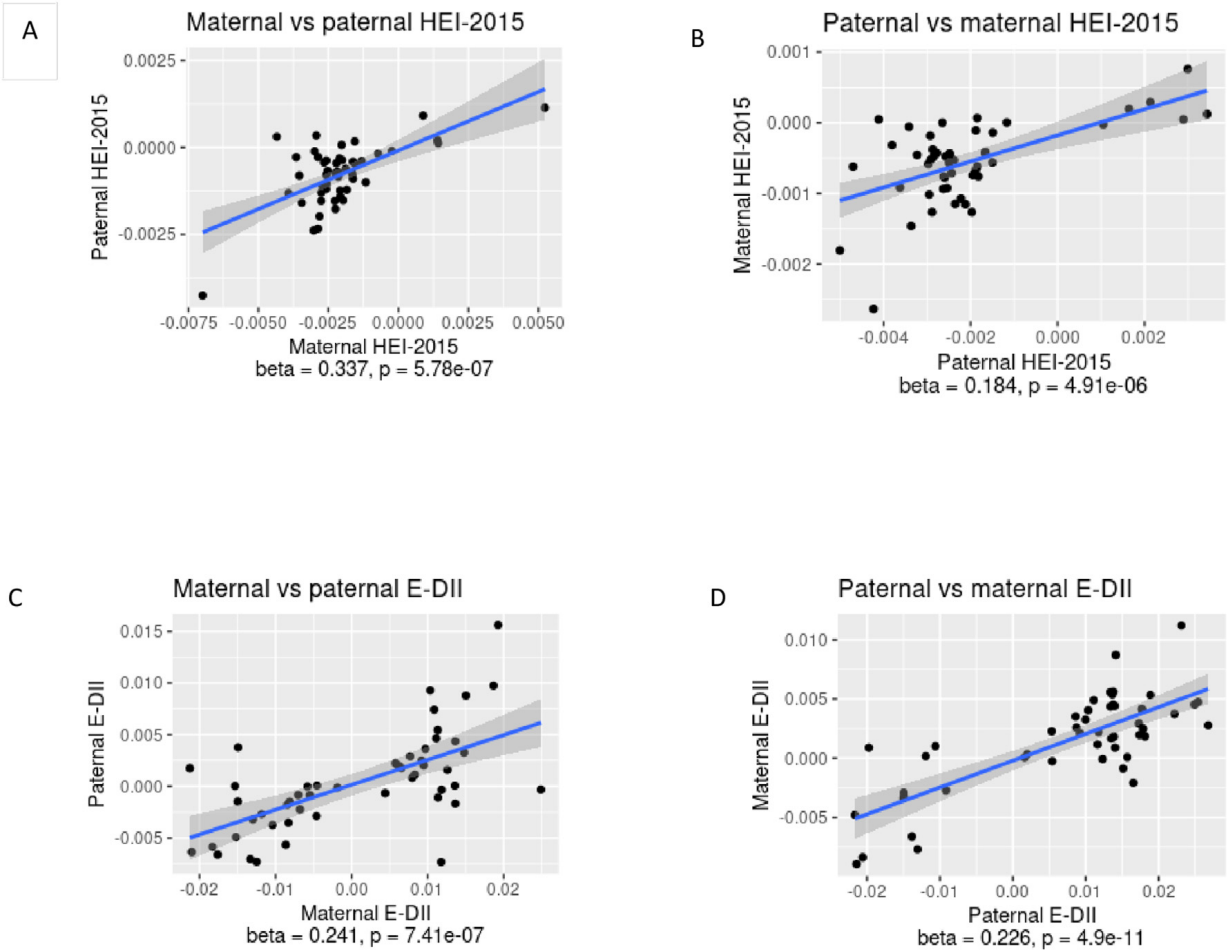
Scatterplots of the comparison of the maternal and paternal dietary associations with individual CpG sites in the Lifeways Cross-Generation Cohort Study. (A) Selection of the top 50 CpG sites associated with the maternal Healthy Eating Index (HEI)-2015, and comparison with the paternal HEI-2015 effects on these CpG sites. (B) Selection of the top 50 CpG sites associated with the paternal HEI-2015, and comparison with the maternal HEI-2015 effects on these CpG sites. (C) Selection of the top 50 CpG sites associated with the maternal energy-adjusted Dietary Inflammatory Index (E-DII), and comparison with the paternal E-DII effects on these CpG sites. (D) Selection of the top 50 CpG sites associated with the paternal E-DII, and comparison with the maternal E-DII effects on these CpG sites. Models adjusted for batch effect, child sex, paternal smoking or maternal smoking, and cellular composition. E-DII, energy-adjusted dietary inflammatory index; HEI, healthy eating index.

Figure 3 shows the comparison of maternal vs paternal effects of the dietary scores on offspring DNAm. At the 50 CpG sites with the strongest associations with maternal HEI-2015, we found similar direction of effects for paternal HEI-2015 (Figure 3A); Similarly, for the 50 CpG sites most strongly associated with paternal HEI-2015, we found a similar direction of effect for associations with maternal HEI-2015 (Figure 3B). Similar findings were noted for the E-DII (Figure 3C, D). Details of the first top 10 CpG sites for each comparison of maternal vs paternal effects on child DNAm are presented in Supplemental Table 6.

#### The HEI-2015 compared with E-DII effect

As expected, given that high values of HEI-2015 indicate increased diet quality whereas high values of E-DII indicate increased diet inflammatory content, inverse direction of effects between the HEI-2015 and E-DII scores were observed. Maternal and paternal HEI-2015 scores were associated with reduced methylation whereas parental E-DII scores were related to high methylation levels (Supplemental Table 7). The correlation between maternal HEI-2015 and E-DII scores was equal to −0.59 (*P* < 2.2 × 10^−16^). Correlation between paternal HEI-2015 and E-DII scores was −0.61 (*P* < 7.5 × 10^−15^). Correlations between maternal and paternal dietary scores were low (*r* = 0.25–0.33) and did not change by marital or living status.

#### DMR analyses

We did not identify any differentially methylated regions for maternal or paternal dietary scores (Bonferroni-adjusted *P* > 0.05).

### Epigenetic age acceleration

**Fig. S1:**
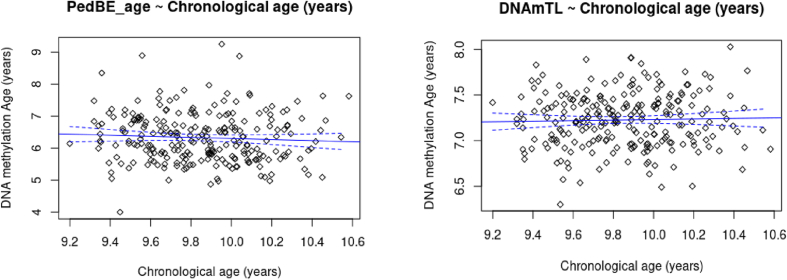
A scatterplot with regression line and 95% CIs showing associations between saliva DNA methylation age derived from PedBE and DNAmTL methods and chronological age in children from the Lifeways birth cohort.

The mean age at saliva sampling was 9.8 y (range: 9.2–10.6), although the mean age estimated using the PedBE method was 6.3 y (range: 4.0–9.3), and 7.3 y (range: 6.3–8.0) for DNAmTL. Correlation between chronological age, the PedBE estimate of age and DNAmTL age was non-significant and close to zero (Pearson’s *r* = −0.06, *P* = 0.33; Pearson’s *r* = 0.03, *P* = 0.66 respectively; Supplemental Figure 1). This low correlation was not unexpected due to the narrow age range in our study (9.2–10.6 y). Similar to other DNAm-based clocks, the median absolute difference between DNAm age and chronological age was 3.6 y. There was however a highly statistically significant negative correlation between the PedBE and DNAmTL estimates (Pearson’s r = −0.39, p = 2.3 ×10^−10^; Supplemental Figure 1). The associations of parental dietary scores and offspring PedBE and DNAmTL acceleration are shown in Table 4. In our study, there was no evidence for associations between any parental dietary scores and PedBE acceleration. Additional adjustment for maternal smoking in paternal models did not alter the associations (Chi-squared test between maternal and paternal smoking = 0.005). In the sensitivity analyses excluding subjects with implausible energy intakes, the effect of the maternal E-DII differed slightly but there was no effect on conclusions for all parental scores (Supplemental Table 8). Similar results were found with DNAmTL estimate. However, using the lower cut-off (3500 kcals), we observed a new association between the maternal DASH score with accelerated telomere aging (β = 0.008, *P* = 0.02) (see Supplementary Table 8).

**TABLE 4:** Associations between parental dietary scores and epigenetic biomarkers of aging in the Lifeways Cross-Generation Cohort Study

Exposure	PedBE age acceleration^1^				DNAmTL acceleration^1^			
	Β^2^ (SE)	*P* value	β^3^ (SE)	*P* value	β^2^ (SE)	*P* value	β^3^ (SE)	*P* value
Maternal dietary scores								
Maternal E-DII	0.02 (0.03)	0.39	0.03 (0.03)	0.38	−0.004 (0.008)	0.67	0.0007 (0.01)	0.94
Maternal HEI-2015	0.001 (0.006)	0.82	0.0002 (0.007)	0.97	0.001 (0.002)	0.56	0.001 (0.002)	0.54
Maternal DASH^4^	−0.002 (0.01)	0.87	−0.006 (0.01)	0.62	0.004 (0.003)	0.17	0.006 (0.004)	0.12
Paternal dietary scores								
Paternal E-DII	0.05 (0.03)	0.15	0.06 (0.041)	0.13	0.007 (0.01)	0.51	0.02 (0.01)	0.18
Paternal HEI-2015	−0.004 (0.006)	0.55	−0.005 (0.007)	0.49	0.002 (0.002)	0.48	0.003 (0.003)	0.24

Data are regression coefficients for an increase in one score unit for each dietary score. *P* < 0.05. ^1^Individual epigenetic age acceleration (y) estimated as the residual from the linear regression of epigenetic age on chronological age, adjusted for the four major estimated cell-types and technical batch (plate). DASH, dietary approach to stop hypertension; E-DII, energy-adjusted dietary inflammatory index; HEI, Healthy Eating Index; SE, standard error. ^2^Adjusted models for child sex and smoking status; maternal scores (*n* = 239), paternal scores (*n* = 123). ^3^Model 1 additionally adjusted for parental age, parity, education level, birthweight, gestational age, and BMI for evaluating associations with maternal dietary scores (*n* = 190), excepted parity for the paternal dietary scores (*n* = 103). ^4^Additional adjustment for energy intake.

Given that DNAmTL was originally derived in blood, and we have applied it in saliva, we sought to evaluate how well DNAmTL in our data might proxy for telomere length. we asked how well associations of DNAmTL in our saliva data resembled published associations between leukocyte telomere length and blood DNAm [15]. Although our EWAS of the adjusted saliva TL did not identify strong associations with DNAm, we observed one association that survived adjustment for multiple tests with the CpG site cg20978460 (p value= 9.6 × 10^−9^), and we did observe consistent direction of effects for the top 30 significant CpG sites associated with leukocyte telomere length (Supplemental Table 9). However, none of these associations survived adjustment for multiple tests (that is, 30 tests).

## Discussion

To our knowledge, this is the first EWAS evaluating whether parental dietary quality in the prenatal period may influence long-term epigenetic variations in children. Higher maternal HEI-2015 scores were inversely associated with DNAm at the CpG site cg21840035 located near the *PLEKHM1* gene. High paternal HEI-2015 scores were associated with reduced methylation at the CpG site cg22431767 located near cell signaling gene *LUZP1*. These associations persisted after adjustments for a range of potential confounders and for multiple testing. Moreover, the findings were robust to sensitivity analysis which was restricted to participants with plausible energy intake by our criteria. Moreover, we did not report any significant associations with the other parental dietary scores or associations between parental dietary quality or dietary inflammation and the DNAm-based estimates of aging in the main analyses. In a sensitivity analysis restricted to women with energy intake >500 and <3500 kcals/d, the maternal DASH scores were associated with decelerated TL shortening.

### Parental dietary associations with offspring DNAm

The two CpG sites associated with the parental HEI scores are located near the genes with distinct functions. The protein encoded by the *PLEKHM1* gene is essential for bone resorption and may play a critical role in vesicular transport in the osteoclast [47]. Mutations in this gene are associated with osteopetrosis in humans, a severe autosomal recessive form occurring *in utero*, infancy, or childhood [48]. The *LUZP1* gene encodes a protein that contains a leucine zipper motif and acts as a cilia regulator which is crucial for cell signaling during development and to maintain organ homeostasis [49]. In animal studies, mutations of this gene may contribute to cardiac malformation and neural tube closure defects in embryos [49,50]. Our findings indicate that parental dietary quality in early development may influence long-term child DNAm. However, no gene expression data was available from the samples to determine whether the methylation changes observed were associated with corresponding changes in the gene expression. Additionally, we caution that microarray probes for some of these CpG sites do coincide with single-nucleotide polymorphisms with global minor allele frequencies >1%; therefore, some DNAm measurements may have been genetically influenced [51]. However, we did not remove these probes before the analysis, as their removal would not much improve *P* value adjustment for multiple tests, and we preferred to maximize the information. No associations between paternal or maternal E-DII scores or maternal DASH scores, and DNAm in the saliva tissue were observed.

There is limited evidence regarding dietary inflammation associations with DNAm. In the Newborn Epigenetics (NEST) study, maternal cytokine concentrations were inversely associated with offspring cord blood methylation of MEG3 DMR but maternal E-DII was not associated with offspring DNAm [52]. However, in that study, almost half of the women had missing FFQ data from the periconceptional period, and only 9 DMRs of biological relevance were examined and paternal diet was not investigated. Although high anti-inflammatory DII scores have been associated with healthier scores for both the DASH and HEI-2010 [53], general dietary quality indices may capture a broader dimension of diet compared with the E-DII. This may, in part, account for our observations with parental HEI-2015 scores. To our knowledge, no other studies have examined parental DASH or HEI-2015 score associations with offspring epigenetics. A high HEI-2015 score reflects increased intake of some food groups that are natural sources of dietary folate (for example, fruits, vegetables, nuts, beans). Folic acid intake is described with increased methylation of *IGF2* in young children that may affect intrauterine growth [54]. PUFAs and MUFAs are also noteworthy HEI-2015 components. Maternal plasma PUFA and SFA concentrations in early pregnancy are related to various changes in newborn cord blood DNAm [55]. Previous analysis of the Lifeways cohort reported that parents with better dietary quality had more favorable characteristics related to smoking status, education level, and income [27,31]. Thus, families with low education or socioeconomic status and potentially poor diet quality, or those with nutritional deficiencies, may be under-represented in our subanalysis, which may partly explain our null findings with E-DII and DASH scores. Further investigation is warranted to understand whether the overall parental dietary quality, evaluated by different approaches, may affect offspring methylation. Differences in dietary assessment, timing of assessment, dietary score ranges, ethnicity, and tissue collected may explain inconsistent results in the literature. However, we did not study the influence of ethnicity in our study, as most of the families had European-born/White ethnicity [11].

### Epigenetic biomarkers of aging

We examined DNAm-derived estimates of aging using the DNAmTL and PedBE models. As our sample had a quite narrow age range (9.2–10.6 y), the correlation between estimated and chronological age was low. Significant technical differences between the DNAm datasets related to how samples were collected and handled, how DNA was extracted, and the specific laboratory conditions when dyes were applied may explain why the average chronological and DNAm-estimated ages differed. However, it is important to note that, despite the technical variation, DNAm age estimates are highly correlated with chronological age, typically R >0.9 when age ranges cover all of childhood or adulthood [56]. DNAmTL exhibited a strong negative correlation with PedBE, consistent with findings for other DNAm-based clocks [46]. Additionally, we showed that the relationship between TL and DNAm is quite different in saliva than in blood. It is possible that the DNAmTL estimate does not actually reflect TL in saliva. Further investigation is required to determine the validity of using DNAmTL as a proxy of TL in saliva.

We did not observe major effects of the parental dietary quality on the DNAm-based estimates of aging. However, our sensitivity analysis showed that a high maternal DASH score was associated with decelerated TL shortening, a well-established biomarker of aging [57]. The early-life dietary factors associated with accelerated DNAm-based estimates of aging remain unknown. Maternal SFA and MUFA intakes during pregnancy were associated with accelerated DNAm age in newborns, whereas vitamin D supplementation was associated with epigenetic age deceleration [13]. Furthermore, a proinflammatory state during pregnancy (assessed by a higher TNF-α/IL-10 ratio) was associated with short offspring TL in newborns [57]. Studies evaluating paternal dietary effects on offspring epigenetics are scarce and focused mainly on the caloric intake [17,19].

### Strengths and limitations

The prospective study design allowed us to examine the effect of early-life exposure to parental diet on the long-term offspring epigenetics. This is important as the prenatal period is a critical window for epigenetic programming because of the major processes that take place at the beginning of pregnancy [58]. Although the epigenome is susceptible to dysregulation throughout life, it is most vulnerable to environmental factors during early development [59].

Despite its strengths, the study does have weaknesses. Information on parental food consumption was self-reported using an FFQ. As this method is memory based and subject to well-known reporting and social desirability biases, it can introduce random error and information bias. Especially, the HEI was developed for use with 24-h recall data, and the use of FFQ data can introduce recall and reporting biases. However, epidemiologic examinations of associations between diet quality and a dependent variable have often relied upon data from FFQs because these are the tools traditionally used in prospective cohort studies [60]. Longitudinal assessment with offspring samples from multiple time points would be relevant to understand long-term stable effects of parental nutrition on offspring DNAm status. However, we did not have epigenetic information during early childhood to assess the stability of these effects.

Furthermore, although the study was sufficiently powered to detect 5% differences in DNAm concentrations (power >80% to detect 5% differences at 90% of 850K CpG sites at Bonferroni-adjusted *P* < 0.05), it was not well-powered to detect small effects [61]. Despite this, our study did uncover some evidence of associations that provides new insights, particularly on the role of the paternal diet. Another unique feature is our focus on epigenetic aging as a relevant biomarker of biological aging and epigenetic health. We used an epigenetic predictor of age specific to children, collected from saliva tissue that was noninvasive, and more feasible in pediatric populations [44]. This method has been used because most other clocks were developed in mainly adult populations whose rate of DNAm change is slower than in pediatric populations [62]. We determined DNAm-derived TL as a distinct measure of biological aging; however, the original estimate was derived in blood. The measure of terminal restriction fragments, the *reference standard* of TL measurements would have been useful to confirm our results [46]. We did not attempt replication because, to our knowledge, there were no other studies with available data on the parental dietary scores and DNAm collected in saliva during early childhood. Finally, as we do not have the data for all relevant family environmental factors (including exposure to passive smoking, child diet), we cannot exclude the possibility of residual confounding by these or unknown factors in pregnancy and during early childhood.

In conclusion, we reported that the parental dietary quality, determined by the HEI-2015 score, in the prenatal period was associated with moderate long-term epigenetic changes in their children. Replication of these results in other studies and examination in heterogeneous populations in terms of diet, for example including populations with nutritional deficiencies or poor dietary quality, is warranted. Improving our understanding of the nutritional programming effects on epigenetic markers is essential to provide scientific arguments for public health strategies that could help parents to achieve a healthier diet and thereby optimize the health capital of future generations. Lastly, other studies of the functional implications of the genes are necessary to understand how they may affect child outcomes.

## Author disclosures

JR Hébert owns controlling interest in Connecting Health Innovations LLC, a company that has licensed the right to his invention of the dietary inflammatory index from the University of South Carolina in order to develop computer and smart phone applications for patient counseling and dietary intervention in clinical settings. The subject matter of this article will not have any direct bearing on that work, nor has that activity exerted any influence on this project.

## Data Availability

The data that support the findings of this study are available from the Lifeways Cross-Generational Cohort Study but restrictions apply to the availability of these data, which were used under license for the current study, and so are not publicly available. Data are however available from the authors upon reasonable request and with permission from the study executive committee.

## Appendix A Supplementary data

Multimedia components 1 Multimedia components 1

Multimedia components 2 Multimedia components 2

Multimedia components 3 Multimedia components 3

Multimedia components 4 Multimedia components 4

Multimedia components 5 Multimedia components 5

Multimedia components 6 Multimedia components 6

Multimedia components 7 Multimedia components 7

Multimedia components 8 Multimedia components 8

Multimedia components 9 Multimedia components 9
