# Supplementary material for: Maternal and Paternal Dietary Quality and Dietary Inflammation Associations with Offspring DNA Methylation and Epigenetic Biomarkers of Aging in the Lifeways Cross-Generation Study
Source: J Nutr. 2023 Jan 28;153(4):1075–88. doi: 10.1016/j.tjnut.2023.01.028 (PMC10196589; doi:10.1016/j.tjnut.2023.01.028)
Supplement: Multimedia components 1 [file mmc1.docx]

Supplemental Table 1: summary of the dietary scores in the Lifeways Cross-Generation Cohort Study

| Dietary scores | Food components and calculation | Interpretation |
| --- | --- | --- |
| Healthy Eating Index 2015  (HEI-2015) (6) | 13 components in total 9 adequacy components:  Total fruits, whole fruits, total vegetables, greens and beans, total protein containing foods, and seafood and plant proteins scored 5 in the highest consumption and 0 in the lowest consumption.  The highest consumption of three components including whole grains, dairy, and fatty acids (ratio of poly- and monounsaturated fatty acids (PUFAs and MUFAs) to SFAs) scored as 10 and the lowest consumption scored as 0.  4 moderate components:  Refined grains, sodium, added sugars, and saturated fats scored 10 in the lowest consumption and 0 in the highest consumption.  Component scores are summed to yield a total score ranging from 0 to 100. | Measure of overall diet quality that measures alignment with the updated 2015-2020 Dietary Guidelines for Americans that are scored on a density basis out of 1000 calories.  A higher score indicating greater adherence to the dietary guidelines for Americans  A HEI score between 51 and 80 is considered as “needing dietary improvement” |
| Energy adjusted Dietary Inflammatory Index (E-DII) (11, 30) | A total of 28 of the 45 possible food parameters were used for DII calculation:  Carbohydrate, protein, fat, alcohol, fibre, cholesterol, saturated fat, mono-unsaturated fat, poly-unsaturated fat, niacin, thiamin, riboflavin, vitamin B12, vitamin B6, iron, magnesium, zinc, selenium, beta-carotene, vitamin A, vitamin C, vitamin D, vitamin E, folic acid, onion, garlic, tea and caffeine.  The food parameter-specific E-DII scores were summed to yield the overall E-DII score | Higher values of the DII indicate a pro-inflammatory (i.e., less healthy) dietary profile, whereas lower values indicate an anti-inflammatory (i.e., more healthy) dietary profile.  The use of the energy-adjusted DII (E-DII) score is more reliable as it takes into account the energy adjustment. |
| Dietary Approaches to Stop Hypertension  (DASH) (8, 29) | 8 food components:  Fruit, vegetables, nuts and legumes, whole grains, low-fat dairy products, sodium, red and processed meats, and sweetened beverages  Participants in the highest quintile received a score of 5 for higher intake of the recommended food components (fruits, vegetables excluding potatoes, total grains, non-full-fat dairy products, and nuts/seeds/legumes), while those in the lowest quintile received a score of 1. Reverse scoring was applied to food components with moderation recommended (red and processed meats, sugar-sweetened beverages/sweets/added sugars, and sodium). | The overall score ranges from 8 (the lowest adherence) to 40 (the highest adherence) |
