# Supplementary material for: Maternal and Paternal Dietary Quality and Dietary Inflammation Associations with Offspring DNA Methylation and Epigenetic Biomarkers of Aging in the Lifeways Cross-Generation Study
Source: J Nutr. 2023 Jan 28;153(4):1075–88. doi: 10.1016/j.tjnut.2023.01.028 (PMC10196589; doi:10.1016/j.tjnut.2023.01.028)
Supplement: Multimedia components 2 [file mmc2.docx]

Supplemental Table 2: Description of EWAS models

1. Methylation ~ maternal dietary score+ child sex +cellular composition +batch effect + maternal smoking (n=244)^1^

Methylation ~ paternal dietary score+ child sex +cellular composition +batch effect + paternal smoking (n=127)

1. Methylation~ model1+ maternal age+ maternal education level+ parity+ birthweight (n=241) ^1^

Methylation~ model1+ paternal age+ paternal education level+ birthweight (n=127)

1. Methylation ~ model2+ gestational age+ maternal BMI (n=194) ^1^

Methylation ~ model2+ gestational age+ paternal BMI (n=108)

^1^DASH score additionally adjusted for energy intake
