# Supplementary material for: Maternal and Paternal Dietary Quality and Dietary Inflammation Associations with Offspring DNA Methylation and Epigenetic Biomarkers of Aging in the Lifeways Cross-Generation Study
Source: J Nutr. 2023 Jan 28;153(4):1075–88. doi: 10.1016/j.tjnut.2023.01.028 (PMC10196589; doi:10.1016/j.tjnut.2023.01.028)
Supplement: Multimedia components 3 [file mmc3.docx]

Supplemental Table 3: Characteristics of Lifeways families followed-up until 10 years with available epigenetic data and those not included in analyses

| Variable | Overall population, N | Lifeways population (n= 825)^1^ | Population with available epigenetic data (n= 259)^1^ | P-value |
| --- | --- | --- | --- | --- |
| Maternal age, y | 1084 | 29.4 ± 6.1 | 32 ± 4.9 | <0.01 |
| Maternal education | 1054 |  |  | <0.01 |
| < Tertiary |  | 435 (54.7) | 100 (38.6) |  |
| ≥ Tertiary |  | 360 (45.3) | 159 (61.4) |  |
| Has medical card | 1071 | 163 (20) | 25 (9.7) | <0.01 |
| Household income > £600 | 967 | 238 (32.7) | 106 (44.4) | 0.0014 |
| Maternal smoking (Yes) | 1083 | 239 (29) | 45 (17.4) | <0.01 |
| Primiparous | 1073 | 376 (46) | 107 (41.8) | 0.24 |
| Paternal age, y | 957 | 32 ± 6.4 | 34.1 ± 5.3 | <0.01 |
| Paternal education | 948 |  |  |  |
| < Tertiary |  | 432 (61.9) | 127 (50.8) | <0.01 |
| ≥ Tertiary |  | 266 (38.1) | 123 (49.2) |  |
| Paternal smoking (Yes) | 325 | 72 (37.7) | 48 (35.8) | 0.73 |
| Maternal delivery | 1023 |  |  | 0.29 |
| Spontaneous |  | 555 (71.3) | 163 (66.5) |  |
| Forceps/ventouse |  | 130 (16.7) | 51 (20.8) |  |
| Cesarean |  | 93 (12) | 31 (12.7) |  |

Values are expressed as mean ± SD and n (%). Chi-squared test used for categorical variables and ANOVA for continuous variables. ^1^Number of families selected, exclusion of the second twin.
