# Supplementary material for: Maternal and Paternal Dietary Quality and Dietary Inflammation Associations with Offspring DNA Methylation and Epigenetic Biomarkers of Aging in the Lifeways Cross-Generation Study
Source: J Nutr. 2023 Jan 28;153(4):1075–88. doi: 10.1016/j.tjnut.2023.01.028 (PMC10196589; doi:10.1016/j.tjnut.2023.01.028)
Supplement: Multimedia components 4 [file mmc4.docx]

Supplemental Table 4: Comparison of the maternal and paternal dietary scores effects on offspring DNA methylation in the different adjusted models

| **Maternal E-DII** | | Model 1 ^1^ | | Model 2 ^2^ | | Model 3 ^3^ | |
| --- | --- | --- | --- | --- | --- | --- | --- |
|  |  |  |  |  |  |  |  |
|  | Probe ID | coefficient1 | p.value1 | coefficient2 | pvalue2 | coefficient3 | pvalue3 |
|  | cg20748132 | 0.015 | 2.28E-07 | 0.0154 | 4.88E-07 | 0.0166 | 1.29E-06 |
|  | cg00109781 | -0.007 | 1.47E-06 | -0.0076 | 1.11E-06 | -0.0074 | 5.51E-06 |
|  | cg13993877 | -0.0153 | 1.47E-06 | -0.0145 | 1.95E-05 | -0.0142 | 1.50E-04 |
|  | cg26871350 | 0.0114 | 2.60E-06 | 0.0126 | 8.15E-07 | 0.0102 | 3.35E-04 |
|  | cg26381263 | -0.0133 | 4.03E-06 | -0.0113 | 1.73E-04 | -0.0103 | 1.97E-03 |
|  | cg22070649 | 0.0095 | 5.05E-06 | 0.0105 | 2.67E-06 | 0.0112 | 1.75E-05 |
|  | cg06708956 | 0.0109 | 6.47E-06 | 0.0117 | 4.27E-06 | 0.011 | 1.40E-04 |
|  | cg01488575 | 0.0083 | 7.73E-06 | 0.0084 | 2.57E-05 | 0.0077 | 6.49E-04 |
|  | cg14336308 | -0.013 | 7.96E-06 | -0.0131 | 2.34E-05 | -0.014 | 8.11E-05 |
|  | cg24284539 | 0.0249 | 8.05E-06 | 0.0268 | 8.38E-06 | 0.0271 | 4.40E-05 |
| **Maternal HEI-2015** | | Model 1 ^1^ | | Model 2 ^2^ | | Model 3 ^3^ | |
|  | Probe ID | coefficient1 | p.value1 | coefficient2 | pvalue2 | coefficient3 | pvalue3 |
|  | cg21840035 | -0.0036 | 5.56E-08 | -0.0038 | 7.41E-08 | -0.0044 | 3.61E-08 |
|  | cg15478184 | -0.003 | 1.10E-07 | -0.003 | 2.83E-07 | -0.0031 | 5.21E-06 |
|  | cg04776779 | -0.0022 | 3.10E-07 | -0.0025 | 3.01E-08 | -0.0025 | 1.88E-06 |
|  | cg01455766 | -0.0039 | 5.27E-07 | -0.004 | 9.82E-07 | -0.0041 | 1.37E-05 |
|  | cg06199676 | -0.0025 | 2.55E-06 | -0.0026 | 4.08E-06 | -0.0022 | 2.60E-04 |
|  | cg22082469 | -0.0021 | 2.91E-06 | -0.002 | 1.25E-05 | -0.0022 | 5.55E-05 |
|  | cg05437285 | -0.0029 | 3.92E-06 | -0.0028 | 1.73E-05 | -0.0026 | 3.75E-04 |
|  | cg00109781 | 0.0014 | 4.57E-06 | 0.0014 | 8.67E-06 | 0.0014 | 7.63E-05 |
|  | cg11468003 | -0.0027 | 5.30E-06 | -0.0029 | 3.22E-06 | -0.0029 | 4.34E-05 |
|  | cg04839673 | -0.0015 | 5.76E-06 | -0.0016 | 1.28E-05 | -0.0014 | 4.24E-04 |
| **Maternal DASH** | | Model 1 ^1^ | | Model 2 ^2^ | | Model 3 ^3^ | |
|  | Probe ID | coefficient1 | p.value1 | coefficient2 | pvalue2 | coefficient3 | pvalue3 |
|  | cg15119693 | -0.0035 | 9.00E-07 | -0.0035 | 6.22E-06 | -0.0035 | 2.0E-04 |
|  | cg10210739 | 0.0023 | 2.55E-06 | 0.0023 | 1.93E-05 | 0.002 | 6.0E-04 |
|  | cg08661219 | 0.0051 | 5.78E-06 | 0.0051 | 2.50E-05 | 0.0052 | 3.0E-04 |
|  | cg20095560 | -0.004 | 9.47E-06 | -0.004 | 6.45E-05 | -0.0037 | 1.3E-03 |
|  | cg20552468 | 0.0054 | 1.41E-05 | 0.0054 | 6.80E-05 | 0.0045 | 3.0E-03 |
|  | cg17859359 | -0.0024 | 1.42E-05 | -0.0024 | 1.04E-04 | -0.0022 | 1.3E-03 |
|  | cg25364619 | -0.0066 | 1.45E-05 | -0.0066 | 1.26E-05 | -0.0071 | 3.0E-04 |
|  | cg22806934 | -0.003 | 1.81E-05 | -0.003 | 1.51E-05 | -0.0036 | 2.75E-05 |
|  | cg17746360 | -0.0075 | 1.91E-05 | -0.0075 | 7.66E-05 | -0.0085 | 2.0E-04 |
|  | cg18045100 | -0.0029 | 2.01E-05 | -0.0029 | 1.99E-05 | -0.0034 | 4.96E-05 |
| **Paternal E-DII** | | Model 1 ^1^ | | Model 2 ^2^ | | Model 3 ^3^ | |
|  | Probe ID | coefficient1 | p.value1 | coefficient2 | pvalue2 | coefficient3 | pvalue3 |
|  | cg16918683 | 0.0178 | 4.59E-07 | 0.0167 | 6.13E-06 | 0.0162 | 1.48E-04 |
|  | cg22431767 | 0.0103 | 6.33E-07 | 0.0103 | 3.31E-06 | 0.0134 | 1.74E-07 |
|  | cg26790423 | 0.0189 | 8.98E-07 | 0.0194 | 4.44E-06 | 0.0206 | 2.09E-05 |
|  | cg20916830 | 0.0268 | 3.40E-06 | 0.0277 | 1.05E-05 | 0.0313 | 8.48E-06 |
|  | cg08287737 | 0.0174 | 3.89E-06 | 0.0151 | 2.00E-04 | 0.0165 | 2.97E-04 |
|  | cg07879720 | 0.0139 | 4.47E-06 | 0.0141 | 2.09E-05 | 0.0155 | 6.65E-05 |
|  | cg16898495 | 0.014 | 7.83E-06 | 0.013 | 1.24E-04 | 0.0146 | 1.38E-04 |
|  | cg24285545 | 0.0086 | 9.79E-06 | 0.0072 | 5.27E-04 | 0.007 | 3.75E-03 |
|  | cg13400365 | 0.0157 | 1.22E-05 | 0.0177 | 5.80E-06 | 0.0177 | 5.52E-05 |
|  | cg13374264 | 0.0138 | 1.32E-05 | 0.0116 | 5.42E-04 | 0.0128 | 1.26E-03 |
| **Paternal HEI-2015** | | Model 1 ^1^ | | Model 2 ^2^ | | Model 3 ^3^ | |
|  | Probe ID | coefficient1 | p.value1 | coefficient2 | pvalue2 | coefficient3 | pvalue3 |
|  | cg22431767 | -0.0022 | 4.12E-08 | -0.0021 | 1.06E-06 | -0.0023 | 8.28E-07 |
|  | cg15311954 | -0.0038 | 3.43E-07 | -0.0041 | 2.69E-07 | -0.0035 | 1.99E-05 |
|  | cg18506400 | -0.0029 | 4.16E-07 | -0.003 | 1.02E-06 | -0.0031 | 3.40E-06 |
|  | cg14977608 | -0.0034 | 4.73E-07 | -0.0034 | 1.67E-06 | -0.0036 | 1.58E-06 |
|  | cg20135776 | -0.0019 | 5.81E-07 | -0.0019 | 2.08E-06 | -0.002 | 2.05E-06 |
|  | cg20595323 | -0.0028 | 9.79E-07 | -0.0029 | 1.51E-06 | -0.0029 | 1.10E-05 |
|  | cg08955721 | -0.0019 | 1.85E-06 | -0.0017 | 2.30E-05 | -0.0019 | 1.21E-05 |
|  | cg14833293 | -0.0029 | 2.05E-06 | -0.0029 | 6.60E-06 | -0.003 | 1.35E-05 |
|  | cg03271761 | -0.0036 | 2.06E-06 | -0.0036 | 1.29E-05 | -0.0035 | 2.75E-05 |
|  | cg25618378 | -0.0041 | 2.22E-06 | -0.0042 | 5.42E-06 | -0.0037 | 1.89E-04 |

DASH: dietary approach to stop hypertension; E-DII: energy adjusted dietary inflammatory index; HEI: healthy eating index

^1^ Selection of the top 10 CpG sites associated with each parental dietary score from model 1. Model1 is adjusted for batch effect, child sex, paternal smoking or maternal smoking and cellular composition.

^2^ Comparison with the effects of each parental dietary score on these CpG sites in model 2. Model 2 is adjusted for previous factors, age, education level, birthweight, and parity (for the maternal model).

^3^ Comparison with the effects of each parental dietary score on these CpG sites in model 3. Model 3 is adjusted for previous factors, in models 1 and 2 and gestational age, BMI at conception.
