# Supplementary material for: Maternal and Paternal Dietary Quality and Dietary Inflammation Associations with Offspring DNA Methylation and Epigenetic Biomarkers of Aging in the Lifeways Cross-Generation Study
Source: J Nutr. 2023 Jan 28;153(4):1075–88. doi: 10.1016/j.tjnut.2023.01.028 (PMC10196589; doi:10.1016/j.tjnut.2023.01.028)
Supplement: Multimedia components 5 [file mmc5.docx]

Supplemental Table 5: Sensitivity analyses – comparison of the maternal and paternal dietary scores effects on offspring DNA methylation after exclusion of subjects with implausible energy intakes

| **Maternal E-DII** | |  |  |  |  |  |  |  |  |  |
| --- | --- | --- | --- | --- | --- | --- | --- | --- | --- | --- |
|  | **Model 1 (n=244)^1^** | | | | **Women > 500 and < 5000 kcal/d (n=238)^1^** | | | **Women > 500 and < 3500 kcal/d (n=223)^1^** | | |
|  | Coefficient | p.value | Chr |  | Coefficient | p.value |  | Coefficient | p.value |  |
| cg20748132 | 0.015 | 2.28E-07 | chr12 |  | 0.0155 | 1.62E-06 |  | 0.0159 | 4.80E-06 |  |
| cg00109781 | -0.007 | 1.47E-06 | chr9 |  | -0.0054 | 3.54E-04 |  | -0.0064 | 9.71E-05 |  |
| cg13993877 | -0.0153 | 1.47E-06 | chr1 |  | -0.0147 | 2.62E-05 |  | -0.0155 | 4.44E-05 |  |
| cg26871350 | 0.0114 | 2.60E-06 | chr5 |  | 0.0139 | 1.33E-07 |  | 0.015 | 1.81E-07 |  |
| cg26381263 | -0.0133 | 4.03E-06 | chr10 |  | -0.0146 | 4.87E-06 |  | -0.0155 | 7.20E-06 |  |
| cg22070649 | 0.0095 | 5.05E-06 | chr2 |  | 0.011 | 1.47E-06 |  | 0.0116 | 2.92E-06 |  |
| cg06708956 | 0.0109 | 6.47E-06 | chr4 |  | 0.0117 | 1.10E-05 |  | 0.0123 | 1.22E-05 |  |
| cg01488575 | 0.0083 | 7.73E-06 | chr8 |  | 0.0095 | 4.05E-06 |  | 0.0108 | 1.43E-06 |  |
| cg14336308 | -0.013 | 7.96E-06 | chr22 |  | -0.0135 | 2.08E-05 |  | -0.0132 | 9.44E-05 |  |
| cg24284539 | 0.0249 | 8.05E-06 | chr10 |  | 0.0203 | 8.07E-04 |  | 0.0196 | 2.50E-03 |  |
|  |  |  |  |  |  |  |  |  |  |  |
| **Maternal HEI-2015** | |  |  |  |  |  |  |  |  |  |
|  | **Model 1 (n=244)^1^** | | | | **Women > 500 and < 5000 kcal/d (n=238)^1^** | | | **Women > 500 and < 3500 kcal/d (n=223)^1^** | | |
|  | Coefficient | p.value | Chr |  | Coefficient | p.value |  | Coefficient | p.value |  |
| cg21840035 | -0.0036 | 5,56E-08 | chr17 |  | -0.0036 | 1.32E-07 |  | -0.0032 | 8.09E-06 |  |
| cg15478184 | -0.003 | 1.10E-07 | chr1 |  | -0.003 | 1.98E-07 |  | -0.0028 | 4.02E-06 |  |
| cg04776779 | -0.0022 | 3.10E-07 | chr10 |  | -0.0023 | 1.66E-07 |  | -0.0023 | 1.28E-06 |  |
| cg01455766 | -0.0039 | 5.27E-07 | chr16 |  | -0.0038 | 2.61E-06 |  | -0.0042 | 1.19E-06 |  |
| cg06199676 | -0.0025 | 2.55E-06 | chr1 |  | -0.0024 | 1.27E-05 |  | -0.0024 | 4.04E-05 |  |
| cg22082469 | -0.0021 | 2.91E-06 | chr12 |  | -0.0021 | 4.68E-06 |  | -0.002 | 3.17E-05 |  |
| cg05437285 | -0.0029 | 3.92E-06 | chr1 |  | -0.0031 | 1.50E-06 |  | -0.0031 | 6.31E-06 |  |
| cg00109781 | 0.0014 | 4.57E-06 | chr9 |  | 0.0013 | 1.71E-05 |  | 0.0013 | 4.16E-05 |  |
| cg11468003 | -0.0027 | 5.30E-06 | chr10 |  | -0.0026 | 1.61E-05 |  | -0.0027 | 2.34E-05 |  |
| cg04839673 | -0.0015 | 5.76E-06 | chr1 |  | -0.0016 | 1.89E-06 |  | -0.0019 | 2.20E-07 |  |
|  |  |  |  |  |  |  |  |  |  |  |
| **Maternal DASH** | |  |  |  |  |  |  |  |  |  |
|  | **Model 1 (n=244)^1^** | | | | **Women > 500 and < 5000 kcal/d (n=238)^1^** | | | **Women > 500 and < 3500 kcal/d (n=223)^1^** | | |
|  | Coefficient | p.value | Chr |  | Coefficient | p.value |  | Coefficient | p.value |  |
| cg15119693 | -0.0035 | 9.00E-07 | chr8 |  | -0.0036 | 8.15E-07 |  | -0.0038 | 1.29E-06 |  |
| cg10210739 | 0.0023 | 2.55E-06 | chr1 |  | 0.0025 | 1.47E-06 |  | 0.0026 | 1.96E-06 |  |
| cg08661219 | 0.0051 | 5.78E-06 | chr5 |  | 0.005 | 1.66E-05 |  | 0.0055 | 3.74E-06 |  |
| cg20095560 | -0.004 | 9.47E-06 | chr19 |  | -0.0042 | 1.05E-05 |  | -0.004 | 4.30E-05 |  |
| cg20552468 | 0.0054 | 1.41E-05 | chr17 |  | 0.0054 | 2.41E-05 |  | 0.0061 | 7.45E-06 |  |
| cg17859359 | -0.0024 | 1.42E-05 | chr1 |  | -0.0026 | 5.96E-06 |  | -0.0028 | 2.64E-06 |  |
| cg25364619 | -0.0066 | 1.45E-05 | chr17 |  | -0.0067 | 2.20E-05 |  | -0.0075 | 3.53E-06 |  |
| cg22806934 | -0.003 | 1.81E-05 | chr11 |  | -0.0029 | 4.67E-05 |  | -0.0029 | 6.50E-05 |  |
| cg17746360 | -0.0075 | 1.91E-05 | chr19 |  | -0.0074 | 4.15E-05 |  | -0.0073 | 1.29E-04 |  |
| cg18045100 | -0.0029 | 2.01E-05 | chr5 |  | -0.003 | 2.22E-05 |  | -0.0031 | 1.39E-05 |  |
|  |  |  |  |  |  |  |  |  |  |  |
| **Paternal E-DII** | |  |  |  |  |  |  |  |  |  |
|  | **Model 1 (n=127) ^1^** | | | | **Fathers > 500 and < 5000 kcal/d (n=126) ^1^** | | | **Fathers > 800 and < 4200 kcal/d (n=123) ^1^** | | |
|  | Coefficient | p.value | Chr |  | Coefficient | p.value |  | Coefficient | p.value |  |
| cg16918683 | 0.0178 | 4.59E-07 | chr15 |  | 0.018 | 4.08E-07 |  | 0.0176 | 1.64E-06 |  |
| cg22431767 | 0.0103 | 6.33E-07 | chr1 |  | 0.0106 | 4.34E-07 |  | 0.0105 | 1.22E-06 |  |
| cg26790423 | 0.0189 | 8.98E-07 | chr2 |  | 0.0191 | 8.66E-07 |  | 0.0186 | 3.52E-06 |  |
| cg20916830 | 0.0268 | 3.40E-06 | chr1 |  | 0.0271 | 3.19E-06 |  | 0.0295 | 9.32E-07 |  |
| cg08287737 | 0.0174 | 3.89E-06 | chr2 |  | 0.0177 | 2.85E-06 |  | 0.0177 | 5.49E-06 |  |
| cg07879720 | 0.0139 | 4.47E-06 | chr5 |  | 0.0141 | 3.40E-06 |  | 0.0151 | 1.57E-06 |  |
| cg16898495 | 0.014 | 7.83E-06 | chr13 |  | 0.0145 | 3.89E-06 |  | 0.0143 | 9.14E-06 |  |
| cg24285545 | 0.0086 | 9.79E-06 | chr2 |  | 0.0085 | 1.39E-05 |  | 0.0081 | 5.03E-05 |  |
| cg13400365 | 0.0157 | 1.22E-05 | chr15 |  | 0.0161 | 7.89E-06 |  | 0.016 | 1.89E-05 |  |
| cg13374264 | 0.0138 | 1.32E-05 | chr1 |  | 0.0141 | 8.73E-06 |  | 0.0133 | 4.52E-05 |  |
|  |  |  |  |  |  |  |  |  |  |  |
| **Paternal HEI-2015** | |  |  |  |  |  |  |  |  |  |
|  | **Model 1 (n=127) ^1^** | | | | **Fathers > 500 and < 5000 kcal/d (n=126) ^1^** | | | **Fathers > 800 and < 4200 kcal/d (n=123) ^1^** | | |
|  | Coefficient | p.value | Chr |  | Coefficient | p.value |  | Coefficient | p.value |  |
| cg22431767 | -0.0022 | 4.12E-08 | chr1 |  | -0.0022 | 4.40E-08 |  | -0.0022 | 1.80E-07 |  |
| cg15311954 | -0.0038 | 3.43E-07 | chr15 |  | -0.0038 | 3.48E-07 |  | -0.004 | 1.58E-07 |  |
| cg18506400 | -0.0029 | 4.16E-07 | chr1 |  | -0.0029 | 3.33E-07 |  | -0.0028 | 9.68E-07 |  |
| cg14977608 | -0.0034 | 4.73E-07 | chr1 |  | -0.0034 | 5.22E-07 |  | -0.0035 | 5.19E-07 |  |
| cg20135776 | -0.0019 | 5.81E-07 | chr1 |  | -0.0019 | 6.35E-07 |  | -0.0018 | 2.51E-06 |  |
| cg20595323 | -0.0028 | 9.79E-07 | chr13 |  | -0.0028 | 1.04E-06 |  | -0.0026 | 7.18E-06 |  |
| cg08955721 | -0.0019 | 1.85E-06 | chr20 |  | -0.0019 | 2.03E-06 |  | -0.0021 | 1.88E-07 |  |
| cg14833293 | -0.0029 | 2.05E-06 | chr22 |  | -0.0029 | 2.26E-06 |  | -0.0029 | 3.29E-06 |  |
| cg03271761 | -0.0036 | 2.06E-06 | chr4 |  | -0.0036 | 2.28E-06 |  | -0.0036 | 3.44E-06 |  |
| cg25618378 | -0.0041 | 2.22E-06 | chr3 |  | -0.0041 | 2.04E-06 |  | -0.0039 | 8.79E-06 |  |

^1^ Selection of the top 10 CpG sites associated with each parental dietary score from model 1. Model1 is adjusted for batch effect, child sex, paternal smoking or maternal smoking and cellular composition; and comparison with the effects of each parental dietary score on the CpG sites in the sensitivity analyses with exclusion of implausible energy intakes by considering a general cutoff of < 500 or >5000 kcal/d, and more gender specific stricter cut offs of < 500 or > 3500 kcal/d for mothers and < 800 or > 4200 kcal/d for fathers.

Chr: chromosome; DASH: dietary approach to stop hypertension; E-DII: energy adjusted dietary inflammatory index; HEI: healthy eating index

Bonferroni significance threshold: 6.3 × 10^−8^
