# Supplementary material for: Maternal and Paternal Dietary Quality and Dietary Inflammation Associations with Offspring DNA Methylation and Epigenetic Biomarkers of Aging in the Lifeways Cross-Generation Study
Source: J Nutr. 2023 Jan 28;153(4):1075–88. doi: 10.1016/j.tjnut.2023.01.028 (PMC10196589; doi:10.1016/j.tjnut.2023.01.028)
Supplement: Multimedia components 6 [file mmc6.docx]

Supplemental Table 6: Comparison of the maternal and paternal dietary associations with individual CpG sites in the Lifeways Cross-Generation Cohort Study

| **Maternal vs paternal HEI-2015^1^** | | | | | |  | | |  |  |  |
| --- | --- | --- | --- | --- | --- | --- | --- | --- | --- | --- | --- |
| Probe ID | | Maternal coefficient | Maternal p value | | | | Paternal coefficient | | Paternal p value | Chromosome | Position |
| cg21840035 | | -0.0036 | 5.56E-08 | | | | -0.0003 | | 0.7372 | chr17 | 43565967 |
| cg15478184 | | -0.003 | 1.10E-07 | | | | -0.0001 | | 0.8889 | chr1 | 248222871 |
| cg04776779 | | -0.0022 | 3.10E-07 | | | | -0.0004 | | 0.4263 | chr10 | 91042527 |
| cg01455766 | | -0.0039 | 5.27E-07 | | | | -0.0013 | | 0.1909 | chr16 | 3211341 |
| cg06199676 | | -0.0025 | 2.55E-06 | | | | -0.0007 | | 0.3376 | chr1 | 9247581 |
| cg22082469 | | -0.0021 | 2.91E-06 | | | | -0.0012 | | 0.0188 | chr12 | 33195036 |
| cg05437285 | | -0.0029 | 3.92E-06 | | | | -0.0023 | | 0.0032 | chr1 | 202644754 |
| cg00109781 | | 0.0014 | 4.57E-06 | | | | 0.0001 | | 0.79 | chr9 | 137936146 |
| cg11468003 | | -0.0027 | 5.30E-06 | | | | -0.0011 | | 0.1683 | chr10 | 23654602 |
| cg04839673 | | -0.0015 | 5.76E-06 | | | | -0.0005 | | 0.2381 | chr1 | 64105555 |
| **Maternal vs paternal E-DII^2^** | | | | | |  | | |  |  |  |
| Probe ID | | Maternal coefficient | Maternal p value | | | | Paternal coefficient | | Paternal p value | Chromosome | Position |
| cg20748132 | | 0.015 | 2.28E-07 | | | | 0.0088 | | 0.0424 | chr12 | 125217707 |
| cg00109781 | | -0.007 | 1.47E-06 | | | | -0.0008 | | 0.7154 | chr9 | 137936146 |
| cg13993877 | | -0.0153 | 1.47E-06 | | | | 2.02E-05 | | 0.9965 | chr1 | 3330724 |
| cg26871350 | | 0.0114 | 2.60E-06 | | | | -0.0011 | | 0.7505 | chr5 | 75919481 |
| cg26381263 | | -0.0133 | 4.03E-06 | | | | -0.0071 | | 0.0553 | chr10 | 133747940 |
| cg22070649 | | 0.0095 | 5.05E-06 | | | | 0.002 | | 0.5095 | chr2 | 18050522 |
| cg06708956 | | 0.0109 | 6.47E-06 | | | | 0.0074 | | 0.0248 | chr4 | 159977429 |
| cg01488575 | | 0.0083 | 7.73 E-06 | | | | 0.0011 | | 0.6694 | chr8 | 1085645 |
| cg14336308 | | -0.013 | 7.96E-06 | | | | -0.0032 | | 0.4088 | chr22 | 50314843 |
| cg24284539 | | 0.0249 | 8.05E-06 | | | | -0.0003 | | 0.9684 | chr10 | 12999599 |
| **Paternal vs maternal HEI-2015^3^** | | | | |  | | |  | |  |  |
|  | | Paternal coefficient | Paternal p value | | | | Maternal coefficient | | Maternal p value | Chromosome | Position |
| cg22431767 | | -0.0022 | 4.12E-08 | | | | -0.0011 | | 0.0044 | chr1 | 23490324 |
| cg15311954 | | -0.0038 | 3.43E-07 | | | | -0.0003 | | 0.6261 | chr15 | 69370499 |
| cg18506400 | | -0.0029 | 4.16E-07 | | | | -0.0005 | | 0.3639 | chr1 | 223418185 |
| cg14977608 | | -0.0034 | 4.73E-07 | | | | -0.0001 | | 0.9272 | chr1 | 55506512 |
| cg20135776 | | -0.0019 | 5.81E-07 | | | | -0.0007 | | 0.0311 | chr1 | 27045762 |
| cg20595323 | | -0.0028 | 9.79E-07 | | | | -0.0004 | | 0.3763 | chr13 | 32470334 |
| cg08955721 | | -0.0019 | 1.85E-06 | | | | 0.0001 | | 0.8433 | chr20 | 10151483 |
| cg14833293 | | -0.0029 | 2.05E-06 | | | | -0.0004 | | 0.4544 | chr22 | 29588618 |
| cg03271761 | | -0.0036 | 2.06E-06 | | | | -0.0009 | | 0.1885 | chr4 | 21699209 |
| cg25618378 | | -0.0041 | 2.22E-06 | | | | 4.76E-05 | | 0.9486 | chr3 | 53753833 |
| **Paternal vs maternal E-DII^4^** | | | | | |  | | |  |  |  |
|  | Paternal coefficient | | | Paternal p value | | Maternal coefficient | | | Maternal p value | Chromosome | Position |
| cg16918683 | | 0.0178 | 4.59E-07 | | | | 0.0041 | | 0.1374 | chr15 | 99276406 |
| cg22431767 | | 0.0103 | 6.33E-07 | | | | 0.0041 | | 0.0225 | chr1 | 23490324 |
| cg26790423 | | 0.0189 | 8.98E-07 | | | | 0.0053 | | 0.1172 | chr2 | 97363955 |
| cg20916830 | | 0.0268 | 3.40E-06 | | | | 0.0028 | | 0.5367 | chr1 | 38583489 |
| cg08287737 | | 0.0174 | 3.89E-06 | | | | 0.002 | | 0.4734 | chr2 | 91932642 |
| cg07879720 | | 0.0139 | 4.47E-06 | | | | 0.0018 | | 0.4355 | chr5 | 37668810 |
| cg16898495 | | 0.014 | 7.83E-06 | | | | 0.0009 | | 0.7023 | chr13 | 80493115 |
| cg24285545 | | 0.0086 | 9.79E-06 | | | | 0.0035 | | 0.035 | chr2 | 180197839 |
| cg13400365 | | 0.0157 | 1.22E-05 | | | | 0.0001 | | 0.972 | chr15 | 86338772 |
| cg13374264 | | 0.0138 | 1.32E-05 | | | | 0.0045 | | 0.0735 | chr1 | 7170661 |

Models adjusted for batch effect, child sex, paternal smoking or maternal smoking and cellular composition. E-DII: energy adjusted dietary inflammatory index; HEI: healthy eating index

^1^ Selection of the top 10 CpG sites associated with the maternal HEI-2015, and comparison with the paternal HEI-2015 effects on these CpG sites.

^2^ Selection of the top 10 CpG sites associated with the maternal E-DII, and comparison with the paternal E-DII effects on these CpG sites.

^3^ Selection of the top 10 CpG sites associated with the paternal HEI-2015, and comparison with the maternal HEI-2015 effects on these CpG sites.

^4^ Selection of the top 10 CpG sites associated with the paternal E-DII, and comparison with the maternal E-DII effects on these CpG sites.
