# Supplementary material for: Maternal and Paternal Dietary Quality and Dietary Inflammation Associations with Offspring DNA Methylation and Epigenetic Biomarkers of Aging in the Lifeways Cross-Generation Study
Source: J Nutr. 2023 Jan 28;153(4):1075–88. doi: 10.1016/j.tjnut.2023.01.028 (PMC10196589; doi:10.1016/j.tjnut.2023.01.028)
Supplement: Multimedia components 7 [file mmc7.docx]

Supplemental Table 7: Comparison of the maternal HEI-2015 and E-DII effects on offspring DNA methylation

| **Maternal HEI-2015 vs E-DII^1^** | | | |  | | **Paternal HEI-2015 vs E-DII^2^** | | | | | |  | |
| --- | --- | --- | --- | --- | --- | --- | --- | --- | --- | --- | --- | --- | --- |
|  | | | |  | |  | |  | |  | |  | |
|  | HEI.coefficient | HEI.p-value | Chromosome | | DII.coefficient |  | HEIcoefficient | | HEI.pvalue | | Chromosome | | DII.coefficient |
| cg21840035 | -0.0036 | 5.56E-08 | chr17 | | 0.0098 | cg22431767 | -0.0022 | | 4.12E-08 | | chr1 | | 0.0103 |
| cg15478184 | -0.003 | 1.10E-07 | chr1 | | 0.0082 | cg15311954 | -0.0038 | | 3.43E-07 | | chr15 | | 0.0125 |
| cg04776779 | -0.0022 | 3.10E-07 | chr10 | | 0.005 | cg18506400 | -0.0029 | | 4.16E-07 | | chr1 | | 0.006 |
| cg01455766 | -0.0039 | 5.27E-07 | chr16 | | 0.0104 | cg14977608 | -0.0034 | | 4.73E-07 | | chr1 | | 0.0073 |
| cg06199676 | -0.0025 | 2.55E-06 | chr1 | | 0.0036 | cg20135776 | -0.0019 | | 5.81E-07 | | chr1 | | 0.0058 |
| cg22082469 | -0.0021 | 2.91E-06 | chr12 | | 0.0077 | cg20595323 | -0.0028 | | 9.79E-07 | | chr13 | | 0.0094 |
| cg05437285 | -0.0029 | 3.92E-06 | chr1 | | 0.0069 | cg08955721 | -0.0019 | | 1.85E-06 | | chr20 | | 0.004 |
| cg00109781 | 0.0014 | 4.57E-06 | chr9 | | -0.007 | cg14833293 | -0.0029 | | 2.05E-06 | | chr22 | | 0.0072 |
| cg11468003 | -0.0027 | 5.30E-06 | chr10 | | 0.0067 | cg03271761 | -0.0036 | | 2.06E-06 | | chr4 | | 0.0128 |
| cg04839673 | -0.0015 | 5.76E-06 | chr1 | | 0.0051 | cg25618378 | -0.0041 | | 2.22E-06 | | chr3 | | 0.0103 |
| cg20165914 | -0.0013 | 6.46E-06 | chr15 | | 0.003 | cg13971652 | -0.0024 | | 2.37E-06 | | chr22 | | 0.0085 |
| cg22073402 | -0.0022 | 6.74E-06 | chr9 | | 0.0064 | cg10162067 | -0.0019 | | 2.69E-06 | | chr20 | | 0.0065 |
| cg12332250 | -0.0026 | 7.13E-06 | chr3 | | 0.0069 | cg27271776 | -0.0026 | | 2.77E-06 | | chr21 | | 0.0057 |
| cg08754124 | -0.0028 | 8.15E-06 | chr16 | | 0.0081 | cg17037045 | -0.0025 | | 2.92E-06 | | chr22 | | 0.0017 |
| cg13022326 | -0.0028 | 9.55E-06 | chr19 | | 0.009 | cg25934995 | -0.0019 | | 3.53E-06 | | chr10 | | 0.0072 |
| cg14645076 | -0.0026 | 1.05E-05 | chr18 | | 0.0047 | cg06313801 | -0.0015 | | 4.44E-06 | | chr17 | | 0.0057 |
| cg02216200 | -0.0022 | 1.07E-05 | chr12 | | 0.0046 | cg18394221 | -0.0029 | | 4.81E-06 | | chr3 | | 0.0055 |
| cg14763036 | -0.0027 | 1.11E-05 | chr3 | | 0.0058 | cg07747690 | -0.0019 | | 5.45E-06 | | chr3 | | 0.0063 |
| cg22159253 | -0.0029 | 1.15E-05 | chr1 | | 0.01 | cg04999558 | -0.0026 | | 5.51E-06 | | chr13 | | 0.0079 |
| cg09084001 | -0.0026 | 1.16E-05 | Chr14 | | 0.0028 | cg09760677 | -0.0026 | | 5.56E-06 | | chr7 | | 0.0064 |

^1^The first 20 highest CpG sites from the maternal HEI-2015 model were selected and compared with the maternal E-DII effect.

^2^The first 20 highest CpG sites from the paternal HEI-2015 model were selected and compared with the paternal E-DII effect.

Model adjusted for batch effect, child sex, parental smoking and cellular composition E-DII: energy adjusted dietary inflammatory index; HEI: healthy eating index
