# Supplementary material for: Maternal and Paternal Dietary Quality and Dietary Inflammation Associations with Offspring DNA Methylation and Epigenetic Biomarkers of Aging in the Lifeways Cross-Generation Study
Source: J Nutr. 2023 Jan 28;153(4):1075–88. doi: 10.1016/j.tjnut.2023.01.028 (PMC10196589; doi:10.1016/j.tjnut.2023.01.028)
Supplement: Multimedia components 8 [file mmc8.docx]

Supplemental Table 8: Sensitivity analyses – associations between parental dietary scores and epigenetic biomarkers of aging after exclusion of subjects with implausible energy intakes

|  | ***PedBE age acceleration^1^*** | | | | ***DNAmTL acceleration^1^*** | | | | |
| --- | --- | --- | --- | --- | --- | --- | --- | --- | --- |
|  |  |  |  |  |  |  |  |  |  |
| **Exposure** | β ^2^ (SE) | P-value | β ^3^ (SE) | P-value | β ^2^ (SE) | P-value | β ^3^ (SE) | P-value | |
| **Maternal dietary scores^4^** |  |  |  |  |  |  |  | |  |
| Maternal E-DII | 0.02 (0.03) | 0.39 | 0.03 (0.03) | 0.38 | -0.004 (0.008) | 0.67 | 0.0007 (0.01) | | 0.94 |
| Maternal HEI-2015 | 0.001 (0.006) | 0.82 | 0.0002 (0.007) | 0.97 | 0.001 (0.002) | 0.56 | 0.001 (0.002) | | 0.54 |
| Maternal DASH^5^ | -0.002 (0.01) | 0.87 | -0.006 (0.01) | 0.62 | 0.004 (0.003) | 0.17 | 0.006 (0.004) | | 0.12 |
| **Exclusion of women with implausible energy intake (< 500 and > 5000 kcal/d)^6^** |  |  |  |  |  |  |  | |  |
| Maternal E-DII | 0.003 (0.03) | 0.92 | -0.002 (0.03) | 0.95 | -0.009 (0.008) | 0.28 | -0.006 (0.01) | | 0.56 |
| Maternal HEI-2015 | 0.002 (0.006) | 0.74 | 0.003 (0.007) | 0.70 | 0.001 (0.002) | 0.43 | 0.001 (0.002) | | 0.54 |
| Maternal DASH^5^ | -0.003 (0.01) | 0.75 | -0.009 (0.01) | 0.52 | 0.005 (0.003) | 0.13 | 0.007 (0.004) | | 0.07 |
| **Exclusion of women with implausible energy intake (< 500 and > 3500 kcal/d)^7^** |  |  |  |  |  |  |  | |  |
| Maternal E-DII | -0.01 (0.03) | 0.68 | -0.02 (0.04) | 0.59 | -0.02 (0.009) | 0.09 | -0.01 (0.01) | | 0.26 |
| Maternal HEI-2015 | 0.001 (0.006) | 0.84 | 0.0006 (0.007) | 0.94 | 0.002 (0.002) | 0.33 | 0.002 (0.002) | | 0.28 |
| Maternal DASH^5^ | -0.004 (0.01) | 0.70 | -0.008 (0.01) | 0.55 | 0.008 (0.003) | **0.02** | 0.001 (0.004) | | **0.008** |
| **Paternal dietary scores^4^** |  |  |  |  |  |  |  | |  |
| Paternal E-DII | 0.05 (0.03) | 0.15 | 0.06 (0.041) | 0.13 | 0.007 (0.01) | 0.51 | 0.02 (0.01) | | 0.18 |
| Paternal HEI-2015 | -0.004 (0.006) | 0.55 | -0.005 (0.007) | 0.49 | 0.002 (0.002) | 0.48 | 0.003 (0.003) | | 0.24 |
| **Exclusion of fathers with implausible energy intake (< 500 and > 5000 kcal/d)^6^** |  |  |  |  |  |  |  | |  |
| Paternal E-DII | 0.05 (0.03) | 0.15 | 0.06 (0.04) | 0.13 | 0.008 (0.01) | 0.47 | 0.02 (0.01) | | 0.18 |
| Paternal HEI-2015 | -0.004 (0.006) | 0.55 | -0.005 (0.007) | 0.49 | 0.002 (0.003) | 0.48 | 0.003 (0.003) | | 0.24 |
| **Exclusion of fathers with implausible energy intake (< 800 and > 4200 kcal/d)^7^** |  |  |  |  |  |  |  | |  |
| Paternal E-DII | 0.05 (0.03) | 0.16 | 0.06 (0.04) | 0.14 | 0.007 (0.01) | 0.55 | 0.02 (0.01) | | 0.18 |
| Paternal HEI-2015 | -0.003 (0.007) | 0.67 | -0.004 (0.007) | 0.60 | 0.001 (0.002) | 0.58 | 0.002 (0.003) | | 0.34 |

^1^ Individual epigenetic age acceleration (years) estimated as the residual from the linear regression of epigenetic age on chronological age, adjusted for the four major estimated cell-types and technical batch (plate). DASH: dietary approach to stop hypertension; E-DII: energy adjusted dietary inflammatory index; HEI: healthy eating index

SE: standard error. Data are regression coefficients for an increase in one score unit for each dietary score.

^2^ Adjusted models for child sex and smoking status

^3^ Model 1 additionally adjusted for parental age, parity, education level, birthweight, gestational age, and BMI for evaluating associations with maternal dietary scores, excepted parity for the paternal dietary models.

^4^ Main analyses model 1: maternal dietary scores (n=239), paternal dietary scores (n=123); model 2: maternal dietary scores (n=190), paternal dietary scores (n=103)

^5^ Additional adjustment for energy intake.

^6^ Exclusion of subjects with implausible energy intakes < 500 or > 5000 kcal/d. model 1: maternal dietary scores (n=230), paternal dietary scores (n=122); model 2: maternal dietary scores (n=182), paternal dietary scores (n=103)

^7^ Exclusion of subjects with implausible energy intakes < 500 or > 3500 kcal/d for mothers and < 800 and > 4200 kcal/d for fathers. Model 1: maternal dietary scores (n=218), paternal dietary scores (n=119); model 2 maternal dietary scores (n=172), paternal dietary scores (n=100) P<0.05.
