# Supplementary material for: Maternal and Paternal Dietary Quality and Dietary Inflammation Associations with Offspring DNA Methylation and Epigenetic Biomarkers of Aging in the Lifeways Cross-Generation Study
Source: J Nutr. 2023 Jan 28;153(4):1075–88. doi: 10.1016/j.tjnut.2023.01.028 (PMC10196589; doi:10.1016/j.tjnut.2023.01.028)
Supplement: Multimedia components 9 [file mmc9.docx]

Supplemental Table 9: Comparison of the adjusted leukocyte TL and saliva TL effects on DNA methylation

| CpG^1^ | Gene | Chr |  |  |
| --- | --- | --- | --- | --- |
|  |  |  | Global meta Z (P) n=5,713^1^ | β (p-value) in saliva n=241^2^ |
| cg08899667 | VARS | 6 | -10.1 (4E-24) | -0.03 (0.21) |
| cg02980249 | VARS | 6 | -8.7 (2E-18) | -0.025 (0.32) |
| cg02597894 | VARS | 6 | -8.1 (4E-16) | -0.031 (0.18) |
| cg04368724 | VARS | 6 | -8.0 (9E-16) | -0.045 (0.15) |
| cg04018738 | VARS | 6 | -8.0 (2E-15) | -0.069 (0.05) |
| cg24771152 | VARS | 6 | -7.8 (6E-15) | -0.037 (0.03) |
| cg20507228 | MAN2A2 | 15 | -9.2 (5E-20) | 0.079 (0.05) |
| cg08972170 | C7orf41 | 7 | -9.0 (2E-19) | -0.019 (0.26) |
| cg27343900 | ERGIC1 | 5 | -8.8 (1E-18) | -0.017 (0.53) |
| cg10549018 | TLL2 | 10 | -8.6 (1E-17) | 0.007 (0.74) |
| cg26709300 | YPEL3 | 16 | -8.6 (1E-17) | -0.07 (0.04) |
| cg27106909 | YPEL3 | 16 | -8.5 (2E-17) | -0.013 (0.3) |
| cg12798040 | XRCC3 | 14 | -8.5 (2E-17) | 0.042 (0.15) |
| cg02194129 | XRCC3 | 14 | -8.3 (1E-16) | -0.014 (0.68) |
| cg19841423 | ZGPAT;LIME1 | 20 | -8.4 (3E-17) | -0.065 (0.04) |
| cg02810967 | NCAPG;DCAF16 | 4 | 8.3 (9E-17) | 0.015 (0.3) |
| cg19935065 | DNTT | 10 | -8.1 (4E-16) | -0.081 (0.04) |
| cg11093760 | CILP | 15 | -8.1 (5E-16) | 0.012 (0.66) |
| cg19097500 | NFIA | 1 | -8.1 (6E-16) | -0.015 (0.14) |
| cg09626867 | EXOSC7 | 3 | -8.1 (7E-16) | 0.023 (0.06) |
| cg04509882 | EIF4G1 | 3 | -8.1 (8E-16) | 0.078 (0.06) |
| cg23661483 | ILVBL | 19 | -8.0 (9E-16) | 0.04 (0.42) |
| cg01012082 | NCOA2 | 8 | -8.0 (1E-15) | -0.007 (0.8) |
| cg21461082 | PRMT2 | 21 | 8.0 (2E-15) | 0.04 (0.37) |
| cg25921609 | MYH10 | 17 | -7.9 (3E-15) | -0.016 (0.7) |
| cg24420089 | PTDSS2 | 11 | -7.8 (8E-15) | 0.021 (0.61) |
| cg07414525 | CHL1 | 3 | -7.8 (9E-15) | 0.048 (0.06) |
| cg14817906 | CNNM4 | 2 | -7.7 (1E-14) | -0.013 (0.36) |
| cg04860432 | PTGER2 | 14 | -7.7 (2E-14) | -0.009 (0.76) |
| cg23570810 | IFITM1 | 11 | 7.7 (2E-14) | 0.07 (0.09) |

**^1^The top 30 most significant CpG sites associated with the fully adjusted leukocyte telomere length (LTL) are shown on the left. Authors conducted an** epigenome-wide association study (EWAS) of adjusted LTL (residuals from a regression of LTL on age and cellular composition) using seven large cohorts (N=5,713) (15).

^2^We calculated a fully adjusted saliva TL as the residuals of a linear regression of DNAmTL estimate on chronological age, adjusted for cellular composition (Buccal, Gran, NK, Bcell). (N=241).
